# A poplar short-chain dehydrogenase reductase plays a potential key role in biphenyl detoxification

**DOI:** 10.1073/pnas.2103378118

**Published:** 2021-08-26

**Authors:** Ángela Contreras, Irene Merino, Enrique Álvarez, David Bolonio, José-Eugenio Ortiz, Luis Oñate-Sánchez, Luis Gómez

**Affiliations:** ^a^Centro de Biotecnología y Genómica de Plantas, Universidad Politécnica de Madrid-Instituto Nacional de Investigación y Tecnología Agraria y Alimentaria, 28223 Pozuelo de Alarcón, Madrid, Spain;; ^b^Environmental Studies Group, Escuela Técnica Superior de Ingenieros de Minas y Energía, Universidad Politécnica de Madrid, 28003 Madrid, Spain;; ^c^Departamento de Sistemas y Recursos Naturales, Escuela Técnica Superior de Ingeniería de Montes, Forestal y del Medio Natural, Universidad Politécnica de Madrid, 28040 Madrid, Spain

**Keywords:** PCB, persistent organic pollutants, phytoremediation, *Populus*

## Abstract

Polychlorinated biphenyls (PCBs) are persistent organic pollutants with severe effects on human health and the biosphere. Plant-based remediation offers many benefits over conventional PCB remediation, but its development has been hampered by our poor understanding of biphenyl metabolism in eukaryotes, among other factors. We report here a major PCB-responsive protein in poplar, a plant model system capable of PCB uptake and translocation. We provide structural and functional evidence that this uncharacterized protein, termed SDR57C, belongs to the heterogeneous short-chain dehydrogenase reductase (SDR) superfamily. Despite sequence divergence, structural modeling hinted at structural and functional similarities between SDR57C and BphB, a central component of the Bph pathway for biphenyl/PCB degradation in aerobic bacteria. By combining gas chromatography/mass spectrometry (GC/MS) profiling with a functional complementation scheme, we found that poplar SDR57C can replace BphB activity in the upper Bph pathway of *Pseudomonas furukawaii* KF707 and therefore catalyze the oxidation of 2,3-dihydro-2,3-dihydroxybiphenyl (2,3-DHDB) to 2,3-dihydroxybiphenyl (2,3-DHB). Consistent with this biochemical activity, we propose a mechanism of action based on prior quantum studies, general properties of SDR enzymes, and the modeled docking of 2,3-DHDB to the SDR57C-NAD^+^ complex. The putative detoxifying capacity of SDR57C was substantiated through reverse genetics in *Arabidopsis thaliana*. Phenotypic characterization of the SDR lines underscored an inducible plant pathway with the potential to catabolize toxic biphenyl derivatives. Partial similarities with aerobic bacterial degradation notwithstanding, real-time messenger RNA quantification indicates the occurrence of plant-specific enzymes and features. Our results may help explain differences in degradative abilities among plant genotypes and also provide elements to improve them.

## Significance

Persistent organic pollutants (POPs), including polychlorinated biphenyls, represent a major environmental threat. Besides affecting human health, they negatively affect food security, pest and disease spread, carbon sequestration, biodiversity, and the resilience of ecosystems. Plant-based remediation offers important advantages over conventional remediation. However, limited knowledge of POP metabolism in planta can delay the application of molecular tools to genetically improve cleanup efficiency. By integrating functional and structural studies, we define here a plant-specific pathway which is activated by and possibly contributes to detoxifying biphenyl-derived toxicants. This pathway exhibits common features with bacterial biphenyl/PCB degradation but also significant differences. Our results open avenues to improve the success of phytoremediation technologies.

Persistent organic pollutants (POPs) are of particular concern due to their blend of toxicity, chemical stability, ability to bio-accumulate, and long-distance transport (1). These compounds pervade food webs even in the most remote habitats and often reach toxic levels in humans and endangered predators (2, 3). Moreover, climatic change and the retreat of sea ice are increasing POP mobilization from natural sinks (4). International regulations are particularly stringent for certain halogenated pesticides and industrial chemicals, most notably polychlorinated biphenyls (PCBs) (1, 2). PCBs are also top-ranked among organic toxicants by the US Agency for Toxic Substances and Disease Registry (https://www.atsdr.cdc.gov/spl), due largely to their toxicity, potential for human exposure, and frequency at facilities on the national priority list.

Remediation of PCB-polluted sites is dominated by energy-intensive conventional technologies (5), but their limited efficiency, ecological disruptiveness, and high costs have sparked the search for innovative solutions (6–8). Phytoremediation is a proven technology that offers important ecological and economic benefits and can be successfully applied in the field under many different conditions (9, 10). Exploitation of its full potential toward organic pollutants is nonetheless hampered by several factors, including our limited understanding of their fate in plants and the roles played by plant-associated microorganisms (6, 7, 9–11). A better molecular knowledge should help implement genomics-based tools to improve pollutant degradation. PCBs are no exception. The only well-defined catabolic pathway for PCBs occurs in a few biphenyl-degrading bacteria isolated from polluted sites (12, 13). These bacteria harbor the *bph* operon, a set of genes conferring the ability to cometabolize biphenyl/PCBs under aerobic conditions. The upper Bph pathway comprises the key oxidative reactions for biphenyl attack, leading sequentially to ring oxygenation, dihydroxylation, and cleavage (12, 13). The second enzyme is the dihydrodiol dehydrogenase BphB, a short-chain dehydrogenase reductase (SDR), which catalyzes the oxidation of the dihydrodiol 2,3-DHDB to the dihydroxy compound 2,3-DHB (12). After ring cleavage by the third enzyme, BphC, the resulting products are metabolized via benzoate and tricarboxylic acid (TCA) cycle intermediates. The fate of chlorine atoms is unclear. Transcriptomic studies in the model species *Arabadopsis thaliana* have focused on lower-chlorinated biphenyls or some hydroxylated derivatives (OH-PCBs), providing the most comprehensive data so far on how plants react to PCBs (14–16). In this respect, protein-based discoveries such as those reported here can add valuable information to transcriptomic approaches. PCBs activate typical multidrug-responsive (MDR) genes, such as those encoding cytochrome P450 (CYP) monooxygenases, glycosyl-transferases, glutathione S-transferases, and ATP-binding cassette transporters. However, a clear-cut connection between enzyme machinery and biphenyl catabolism has yet to emerge in eukaryotes. In plants, similar gene expression profiles are induced by PCBs and safeners (16, 17), a heterogeneous set of nonbiphenyl compounds used to protect crops against pesticides and herbicides. Likewise, homologous MDR genes are induced in humans and other mammals by PCBs (18) but also by structurally unrelated drugs (19).

Within the plant kingdom, the “tree” phenotype offers many intrinsic advantages to deal with pollution (10). In this respect, poplars (genus *Populus*) have proven particularly apt for remediating a wide array of pollutants under field settings, including PCBs (9–11, 20, 21). Furthermore, *Populus* has long been recognized as a model system (22). By challenging poplar plants with PCB mixtures or biphenyl, we identify a major responsive enzyme which may be pivotal for biphenyl detoxification. We show that it is a member of the SDR superfamily, one of the most heterogeneous and less functionally understood groups of plant enzymes (23, 24). Structural and functional evidence is presented documenting the oxidizing activity of the novel enzyme toward 2,3-DHDB, a central metabolite of the upper Bph pathway in bacteria. Additional biphenyl-responsive enzymes were identified which can participate in biphenyl degradation. These plant enzymes show little or no sequence similarity with their bacterial “counterparts.” Taken together, our results indicate the existence of a plant-specific pathway for biphenyl/PCB catabolism.

Ectopic overexpression of poplar SDR57C in *Arabidopsis* supports its putative detoxifying capacity and may explain differences between *Arabidopsis* and poplar when grown in the presence of biphenyls. Our findings challenge the notion of a modern origin for biphenyl catabolism, thought to be triggered by the bulk amounts of PCBs released by industry into the environment.

## Results and Discussion

### SDR57C Is a Major PCB-Responsive Protein in Poplar.

**Fig. 1.**
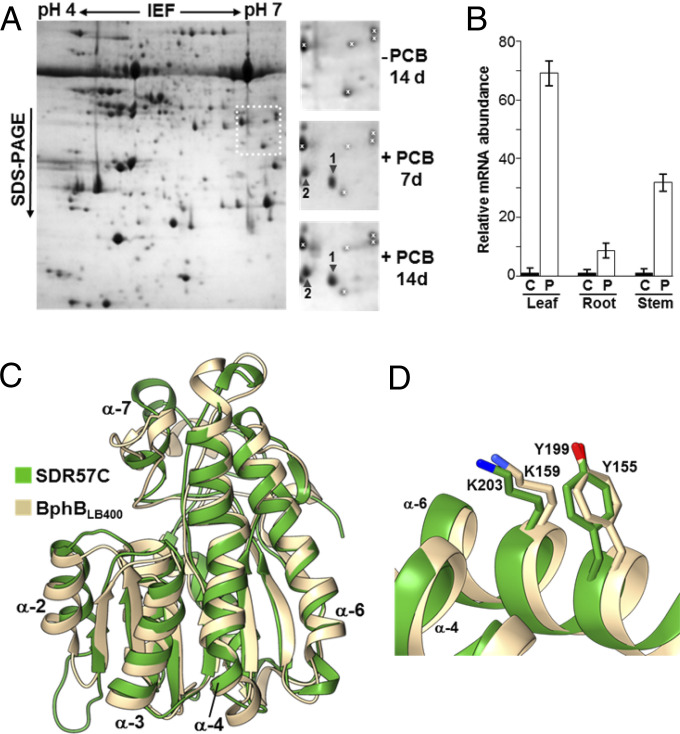
Major PCB-responsive enzyme in hybrid poplar. (*A*) Coomassie-stained leaf proteins after IEF × SDS-PAGE. Plants were exposed for up to 14 d to DMSO (−PCB controls) or to 100 mg/L of Aroclor 1221 (+PCB). DMSO concentration was identical in all cultures (0.1%, vol/vol). A complete map (two leaves from four control plants, pooled) is shown on the left. In contrast to control samples, two major proteins (arrowheads) were observed at ∼33 kDa in PCB-exposed samples (two biological replicas). They were later identified by mass fingerprinting (*SI Appendix*, Table S1). White crosses sign reference spots. (*B*) Transcript abundance for SDR57C as measured by qRT-PCR. C, control (DMSO-exposed) plants. P, PCB-exposed plants. Mean values of control samples were used to index data. Error bars represent SEMs (*n* = 3; two leaves from four plants per replica). (*C*) Structural alignment of *P. tremula* SDR57C (green) and *B. xenovorans* BphB_LB400_ (gray; PDB code: 1BDB). (*D*) Zoom at the active site of BphB_LB400_. Its catalytic residues Y155 and K159 are closely matched by Y199 and K203 in the poplar SDR57C model. The intervening residues are not conserved.

Accumulation of two major responsive leaf polypeptides was consistently detected in *Populus tremula* x *Populus alba* plants exposed to PCB mixtures but not in plants exposed to the PCB solvent (dimethyl sulfoxide, DMSO) alone (Fig. 1*A*). Matrix-assisted laser desorption/ionization time-of-flight (MALDI-TOF) mass spectrometry analyses (*SI Appendix*, Table S1) followed by Mascot searches (only feasible for *Populus trichocarpa*) uncovered a single protein fitting the mass fingerprint data in *P. tremula* and *P. alba* (*SI Appendix*, Fig. S1). Conserved motifs place these proteins into the SDR57C subfamily of “classical” SDRs (24), formed by a single member in the three genomes considered here. Based on the MS data, poplar plants were analyzed by qRT-PCR with specific primers (*SI Appendix*, Table S2). Compared to transcript abundance in DMSO controls, PCB exposure (100 mg/L) caused significant *SDR57C* activation in all organs analyzed, mainly in leaves (Fig. 1*B*). Whereas sequences in this SDR subfamily have been associated with glucose/ribitol dehydrogenases (23), their activity in plants is unknown. To gain functional insight, we identified the closest proteins in *P. trichocarpa*, the best annotated *Populus* genome to date. Given the remarkable heterogeneity of plant SDRs (23, 24), identities down to 25% were considered as long as query coverage was 85% or superior. The ensuing clustering analysis (*SI Appendix*, Fig. S2) placed poplar SDR57C closest to NADPH-dependent aldehyde reductases (68 to 69% identity to *P. tremula* SDR57C, 98% coverage). Both groups are loosely related to 11-β-hydroxysteroid dehydrogenases (29% identity, 88% coverage). The remaining components in the phylogram were SDR enzymes acting on substrates as diverse as xanthoxin, secoisolariciresinol, 3-oxoacyl-acyl-carrier-protein, tropinone, 2,4-dienoyl-CoA, or isopiperitenol/carveol. A shared structural motif cannot be envisioned from these substrates.

To investigate structure–function relationships, a structural model for SDR57C was generated with SWISS-MODEL (277 residues; *SI Appendix*, Fig. S3*A*). This server automatically selected as top template a crystallized SDR-NAD^+^ complex from *Bacillus anthracis* (Protein Data Bank [PDB] code 3I3O; 52% identity). The predicted secondary structure elements (*SI Appendix*, Fig. S3*B*) defined a typical Rossmann-fold motif for dinucleotide binding: a central, twisted parallel β-sheet (topology 3–2-1-4-5-6-7) sandwiched by eight α-helices. Docking simulations followed by energy minimization provided insight into the putative coenzyme binding site (*SI Appendix*, Fig. S4*A*). Intermolecular hydrogen bonds and hydrophobic interactions can be predicted to stabilize the binary complex. The presence of Asp52 at the Gly-rich region next to strand 1 should favor binding of NAD^+^ over NADP^+^, as the carboxylate would lie too close to the extra phosphate and the side chain of Tyr74 would clash with it. The presence of a charged residue at an analogous position dictates the NAD^+^/NADP^+^-dependence of other SDRs (25). Strikingly similar geometries have been reported for NAD^+^ coenzymes crystallized in complex with phylogenetically distant SDR enzymes (*SI Appendix*, Fig. S4*B*). Their empirical structures were not used as templates in this project. We searched for proteins structurally analogous to our optimized complex using PDBeFold and mTM-align. Both engines identified a few bacterial enzymes associated with xenobiotic degradation: *cis*-2,3-DHDB dehydrogenases from *Burkholderia xenovorans* (syn. *Pseudomonas*) sp. LB400 (PDB code: 1BDB) and *Pandoraea pnomenusa* strain B-356 (PDB codes: 2Y93, 2Y99, and 3ZV3-3ZV6) as well as a *cis*-dihydrodiol naphthalene dehydrogenase from *Pseudomonas* sp. MC1 (PDB code: 5XTG). Except 5XTG, the remaining enzymes are involved in biphenyl catabolism. The Bph pathway has been thoroughly investigated in several PCB degraders, mainly *Pseudomonas furukawaii* (syn. *P. pseudoalcaligenes*) KF707, *B. xenovorans* LB400, and *Acidovorax* (syn. *Pseudomonas*) sp. KKS102 (12, 13). These enzymes are encoded by *bphB* genes and catalyze the NAD^+^-dependent oxidation of 2,3-DHDB to 2,3-DHB. Fig. 1*C* shows the structural alignment of SDR57C and the best hit found by PDBeFold, namely, BphB_LB400_ (PDB code 1BDB, chain A). The spatial resemblance of both apoenzyme cores is astounding (RMSD of 1.07 Å; 182 C-α pairs) for an overall sequence identity of only 30% (*SI Appendix*, Fig. S5). Identical residues are located mainly in conserved core regions (boxed in *SI Appendix*, Fig. S1), while peripheral loops exhibit more divergence. The catalytic residues of BphB_LB400_, Tyr155, and Lys159, are spatially matched in SDR57C by Tyr199 and Lys203 (Fig. 1*D*). These findings strongly suggest that poplar SDR57C may directly attack biphenyl moieties in the same manner as BphB enzymes.

### Functional Replacement of BphB Activity.

**Fig. 2.**
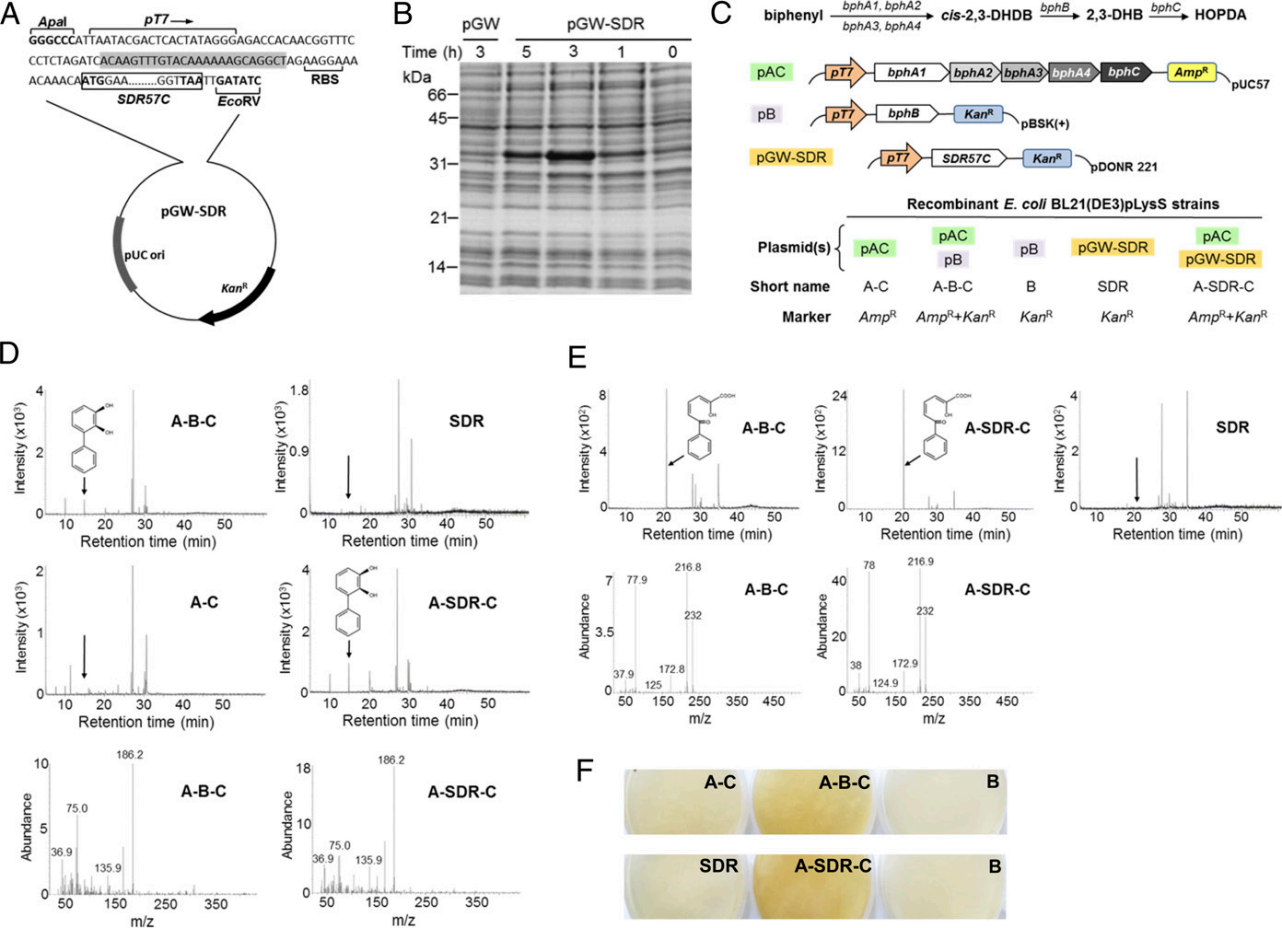
Expression in *E. coli* of poplar SDR57C and selected Bph enzymes from *P. furukawaii* KF707. (*A*) The coding sequence of SDR57C controlled by the *T7* promoter (*pT7*) of pDEST14 was cloned into Gateway pDONR 221, giving rise to pGW-SDR. The 9-bp sequence upstream of the start codon is coincident with the poplar gene. RBS, ribosome-binding sequence. Gray shading, partial *att*B1 site generated after LR recombination with pDEST14. (*B*) SDS-PAGE fractionation of soluble bacterial proteins (∼25 μg) from IPTG-induced *E. coli* BL21(DE3)pLysS cultures. Cells were transformed with pGW-SDR or pGW. After IPTG induction, aliquots were taken at the times indicated, and bacterial proteins were analyzed. (*C*, *Top*) Upper Bph pathway and plasmid constructs for bacterial protein expression (refer to main text). (*Bottom*) The indicated recombinant strains were obtained to test the interchangeability of poplar SDR57C and BphB. (*D*) 2,3-DHB detection in whole-cell assays, 5 h after biphenyl addition. (*Upper*) Extracted ion chromatograms at m/z 186 for the indicated samples. (*Lower*) Mass spectra of the 2,3-DHB peak detected in A-B-C (positive control) and A-SDR-C cultures. 2,3-DHB was not detected in negative controls (i.e., A-C, SDR, or B cultures) (A-C and SDR are shown). mAU, milli-absorption units. (*E*) Detection of HOPDA in identical whole-cell assays. (*Upper*) Extracted ion chromatograms at m/z 232 for the indicated samples. (*Lower*) Mass spectra of the HOPDA peak detected in A-B-C (positive control) and A-SDR-C cultures. HOPDA was not detected in negative controls (SDR is shown). (*F*) Visual detection of HOPDA in inoculated LB-agar plates containing biphenyl (300 mg/L) and IPTG (0.5 mM).

To test the hypothetical activity of SDR57C, we attempted the functional complementation of a Tn-insertion Δ*bphB* mutant generated in *Acidovorax* sp. KKS102 (26) (*SI Appendix*, Fig. S6*A*). This strategy did not work, however, because the poplar protein was proteolytically inactivated (*SI Appendix*, Fig. S6 *B–*D). Since different subsets of *bph* genes have been successfully expressed in *Escherichia coli* (e.g., refs. 27, 28), we designed a functional complementation scheme for this host. The coding sequence of SDR57C was thus placed under the control of a *T7* promoter (p*T7*) in pGW-SDR (Fig. 2*A*). The same backbone was opened, blunted, and self-ligated to render pGW, and both plasmids were transformed into *E. coli* BL21(DE3) pLysS. After isopropyl-β-D-thiogalactopyranoside (IPTG) induction, a ∼32-kDa band was detected only in protein extracts from pGW-SDR cultures (Fig. 2*B*). Its relative intensity was maximal about 3 h after induction, and its apparent size was the expected for native SDR57C. Based on the upper Bph pathway, which converts biphenyl into 2-hydroxy-6-oxo-6-phenylhexa-2,4-dienoic acid (HOPDA) (Fig. 2 *C*, *Top*), two additional expression plasmids were constructed carrying specific *bph* genes from *P. furukawaii* KF707: 1) plasmid pAC, a pUC57 backbone (*Amp*^*R*^) harboring the upper Bph pathway genes *bphA* (*A1*, *A2*, *A3*, and *A4*) and *bphC* under the control of p*T7*; and 2) plasmid pB, a pBSK(+) backbone (*Kan*^*R*^) harboring the *bphB* gene controlled by the same promoter. The complete cloned sequences are described in *SI Appendix*, Fig. S7 (pAC) and *SI Appendix*, Fig. S8 (pB). To test the interchangeability of BphB and SDR57C, five recombinant *E. coli* BL21(DE3)pLysS strains were obtained (Fig. 2 *C*, *Bottom*). We chose a pLysS host due to the toxicity exhibited by biphenyl catabolites in preliminary induction experiments with *E. coli* BL21, in line with ref. 27. pLysS reduces background expression by constitutively producing lysozyme, a specific inhibitor of the T7 RNA polymerase. Besides the pLysS block, induction conditions were adjusted to reduce toxicity. Whole-cell assays followed by gas chromatography/mass spectroscopy (GC/MS) analysis revealed that recombinant A-B-C and A-SDR-C cells were able to convert biphenyl into 2,3-DHB (Fig. 2*D*), the expected product of BphB activity (12). This catechol was not detected in quantitatively comparable extracts from A-C, B or SDR cells (negative controls). Consistent with these findings, HOPDA was identified by GC/MS only in A-SDR-C and A-B-C cells (Fig. 2*E*). HOPDA is the yellow metacleavage product of BphC acting on 2,3-DHB (Fig. 2*C*). HOPDA was visually observed when A-B-C or A-SDR-C cells grew on Luria-Bertani (LB) agar containing biphenyl and IPTG but not in comparable plates inoculated with A-C, B or SDR cells (Fig. 2*F*) or lacking IPTG. Taken together, these findings demonstrate that SDR57C and BphB_KF707_ catalyze the same reaction in the engineered upper Bph pathway and hence substantiate the capacity of the poplar enzyme to oxidize 2,3-DHDB to 2,3-DHB. To our knowledge, dehydrogenation of a dihydrodiol biphenyl skeleton has not been described before for a eukaryotic enzyme.

### Studies *In Planta*.

**Fig. 3.**
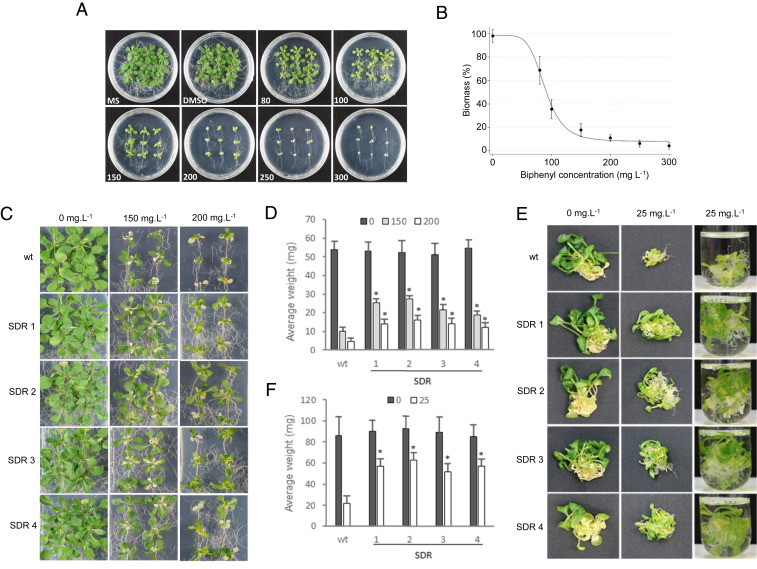
Toxic effects of biphenyl on *A. thaliana*. (*A*) Representative photograph of wt seedlings after 17-d exposure to DMSO, biphenyl in DMSO (biphenyl concentrations are in mg/L), or neither (MS control). (*B*) Dose–response curve as determined through growth tests. Biomass is expressed relative to DMSO-exposed samples. The solid line was fitted to a four-parameter logistic function with Stata (*R*^2^ = 0.98). Error bars represent SDs between four biological replicates. (*C*) Growth in solid medium of wt and T4 transgenic lines exposed to the indicated doses of biphenyl for 17 d. (*D*) Averaged biomass production in solid medium (*C*) of four biological replicas (*n* = 4; 5 × 6 plants per line and replica; 6 plants per plate). (*E*) Growth in liquid medium of wt and T4 transgenic lines exposed to biphenyl for 13 d. (*F*) Averaged biomass measurements in liquid medium (*E*) at a biphenyl concentration of 25 mg/L (*n* = 4; 5 × 7 plants per line and replica). Asterisks indicate significant (*P* < 0.05) differences between SDR and wt lines as determined by Student’s *t* tests. Except in the MS samples (*A*), 0.1% (vol/vol) DMSO was present in all growth media.

**Fig. 4.**
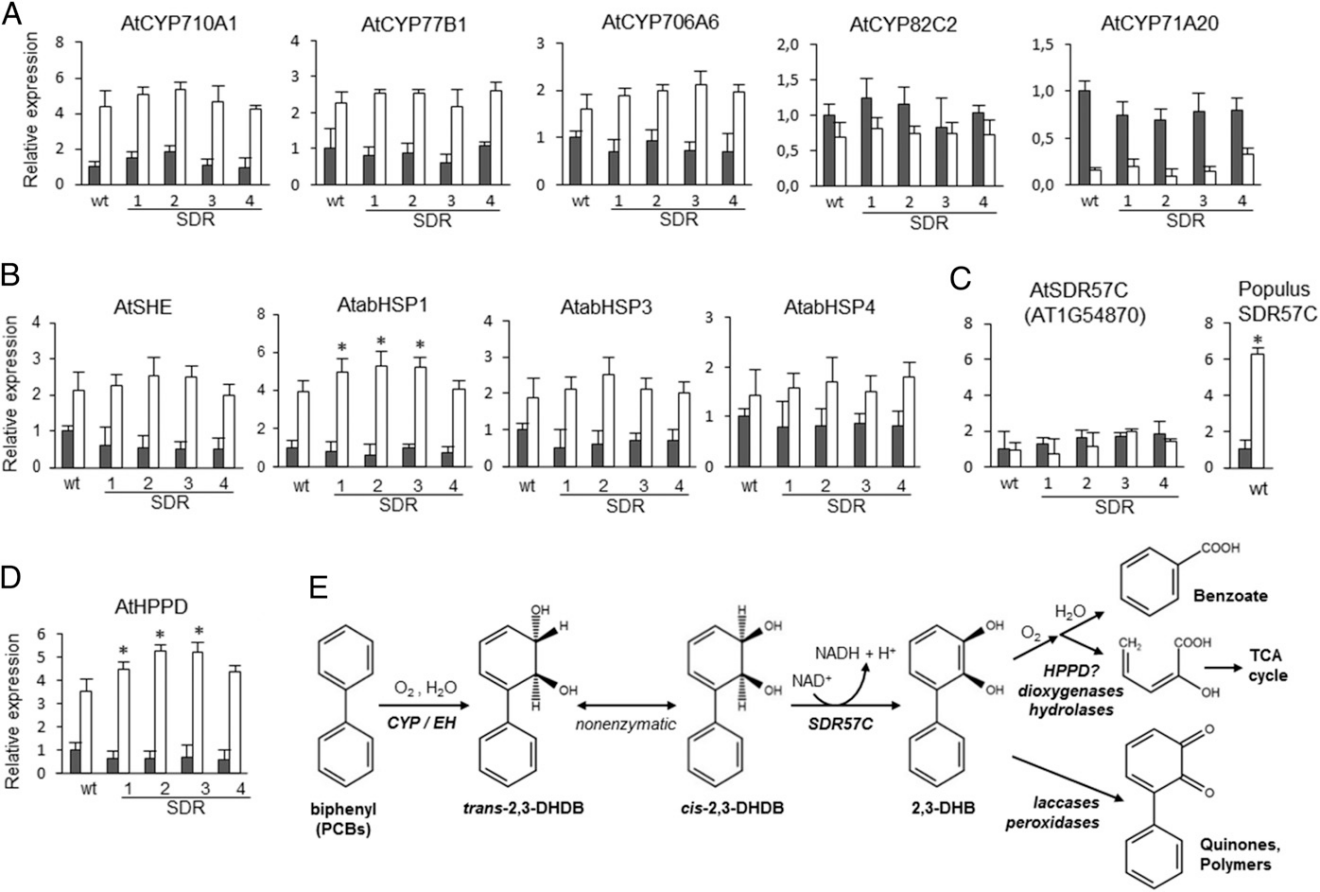
Biphenyl-responsive genes and proposed detoxifying pathway. After exposure to biphenyl (150 mg/L, white bars) or DMSO (black bars) for 5 d in solid medium, relative transcript abundance (fold change) was measured by qRT-PCR in *A. thaliana* wt and SDR lines. Primers and gene IDs are listed in *SI Appendix*, Table S2. Data were normalized to *ACT8*. Mean values for DMSO-exposed wt samples were used to index data. SEMs were calculated from three biological replicates (four pooled plants per line and replica). Asterisks indicate significant (*P* < 0.05) differences between SDR and wt lines as determined by Student’s *t* tests. (*A*) CYP genes. (*B*) EH genes. (*C*) SDR57C genes. Transcript abundance in poplar was determined after 12 d exposure to biphenyl (150 mg/L), and data were normalized to*18S rRNA*. (*D*) HPPD gene. (*E*) Proposed plant pathway for biphenyl detoxification. Quinone formation is a plausible alternative to catechol ring-cleavage (18, 30). Nearly identical results were obtained when *Arabidopsis* data were normalized to *18S rRNA* or *Populus* data to *UBQ7*.

The experimental evidence that SDR57C is able to oxidize 2,3-DHDB prompted us to investigate its putative detoxifying role in planta. To this end, we analyzed the toxicity of biphenyl for *A. thaliana*, an aspect for which previous information was not available. We used axenic conditions to prevent microbial interference. Due to the low polarity of biphenyl, concentrated solutions in DMSO were dispersed into culture media so that 0.1% (vol/vol) DMSO was present in all cultures. The growth assays on agar plates uncovered toxic effects for biphenyl but not for DMSO at the levels tested (Fig. 3 *A* and *B*). A half-maximal (50%) inhibitory concentration (IC_50_) of 89.0 mg/L was estimated from the dose–response data (Fig. 3*B*). Several PCBs or OH-PCBs have been evaluated in comparable assays (15, 16), with IC_50_ estimates ranging from 1.6 mg/L for 4′-OH-4-chlorobiphenyl to 12 mg/L for 4-chlorobiphenyl. Yet, no toxic effects were observed for some congeners (e.g., 2,5-dichlorobiphenyl) at concentrations of 100 mg/L (16). We overexpressed poplar SDR57C in *A. thaliana* via stable transformation, using the CaMV *35S* promoter harbored by the binary vector pB2GW7 (*SI Appendix*, Fig. S9). Plant growth was then analyzed upon biphenyl exposure. A minimal concentration of 150 mg/L was selected for quantitative assays in agar plates because it caused in wild-type (wt) plants a significant impact on biomass production (ca. 80% reduction after 17 d; Fig. 3*B*) relative to nonexposed or DMSO-exposed controls. Whereas DMSO had no measurable effects on plant growth, biphenyl exposure caused substantial differences between T4 transgenic lines and wt controls (Fig. 3 *C* and *D*). On average, at 150 mg/L, the SDR lines exhibited fresh weights after 17 d up to 2.7-fold higher than controls (compare wt with SDR1 or SDR2 lines in Fig. 3*D*). In a different set of experiments, growth was monitored under hydroponic conditions, as they promote root development and whole-plant contact with the toxicant. Significant differences were recorded again between wt and SDR lines after exposure to biphenyl but not to DMSO (Fig. 3 *E* and *F*). These results were suggestive of a detoxifying in vivo role for SDR57C, most likely through its catalytic activity toward dihydrodiol biphenyl. In the face of sequence divergence, the striking similarities between SDR57C and bacterial BphB pointed at a similar biochemistry in plants and aerobic biphenyl/PCB-degrading bacteria. The first bacterial step is conversion of biphenyl to 2,3-DHDB by the biphenyl dioxygenase system (*bphA* genes in Fig. 2*C*). However, we did not find homologous genes in *Arabidopsis* or any description of such conversion in plants (e.g., refs. 7, 8). Vicinal arene dihydrodiols are formed in eukaryotes mainly via epoxide intermediates, through the sequential action of CYP epoxidases and epoxide hydrolases (EHs) (18, 29, 30). Since activation (and repression) of CYP gene subsets in response to PCBs or OH-PCBs had been documented in transcriptomic studies of *A. thaliana* (14–16), we analyzed the activity of the most up-regulated genes (mostly approximately two- to fourfold relative to controls) in our plant material. Biphenyl exposure enhanced transcript abundance for AtCYP77B1, AtCYP710A1, and AtCYP706A6 in all lines analyzed (Fig. 4*A*). In contrast, transcripts for AtCYP82C2 and AtCYP71A20 diminished, remaining unchanged for AtCYP707A2. Epoxidase activity is thought to be a general property of CYPs (29, 30) and has been specifically demonstrated for the CYP77 clan (31). The other biphenyl-induced clans have been associated to arene metabolism −CYP710A to sterol biosynthesis (32) and the functionally promiscuous CYP706A to dinitroaniline herbicide tolerance (33). By analyzing the same *Arabidopsis* material with appropriate primers (*SI Appendix*, Table S2 and Fig. S10), biphenyl-induced activation was recorded for three EH genes, with *abHSP1* (AT4G15960.1) showing the strongest response (Fig. 4*B*). Although EHs have been barely characterized in plants (34), the CYP/EH action on biphenyl is expected to generate 2,3-DHDB, a fate proposed in mammalian systems (18). SDR57C was shown here to oxidize 2,3-DHDB to 2,3-DHB (Fig. 2), although other substrates cannot be ruled out (35). Interestingly, biphenyl had no appreciable effects on the activity of endogenous *SDR57C* in *A. thaliana* (Fig. 4*C*), in sharp contrast with the activating effects recorded in poplar plants exposed to PCBs (Fig. 1*B*) or biphenyl itself (Fig. 4*C*). Such differences provide a direct explanation for the superior performance of SDR lines over controls upon biphenyl challenge (Fig. 3). After 2,3-DHB formation, ring opening by a BphC-like activity would complete the upper catabolic pathway (12). Although no BphC homologs were found in *A. thaliana*, we used the atomic coordinates for BphC_KKS102_ (PDB code: 1KW3) to identify structural analogs. Both mTM-align and PDBeFold retrieved a single matching structure of plant origin: *A. thaliana* 4-hydroxyphenylpyruvate dioxygenase (AtHPPD; PDB code: 1SQD) (36), an enzyme involved in aromatic amino acid catabolism. Sequence divergence notwithstanding, the architecture of both active sites is remarkably well conserved (36), with an RMSD of only 1.0 Å for a “core” of 80 C-α pairs. Although such similarity does not prove functional equivalence, the finding that *AtHPPD* transcription is significantly activated in wt and SDR lines by biphenyl exposure (Fig. 4*D*) remains an intriguing observation worthy of further research. A plant catabolic pathway based on the preceding discussion is proposed in Fig. 4*E*. Additional work will be needed to clarify the 2,3-DHDB isomers metabolized by SDR57C. While trans isomers can be expected from CYP/EH action (18, 37), a low-energy barrier has been estimated for 2,3-DHDB racemization (38). Spontaneous racemization has been documented for other arene dihydrodiols (37). By either path, the product of SDR57C is the nonchiral catechol 2,3-DHB (Fig. 4*E*), more amenable to biodegradation than biphenyl (6–8, 12).

### Substrate Binding and Mechanism of Biphenyl Attack.

**Fig. 5.**
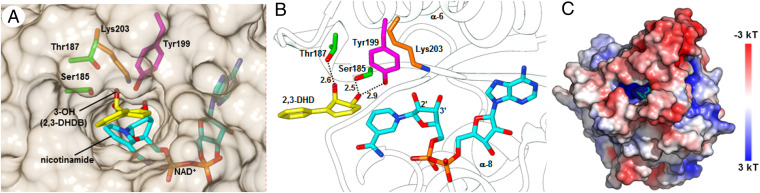
Modeled docking of 2,3-DHDB to the SDR57C–NAD^+^ complex. (*A*) Front view of the substrate binding pocket, showing the local geometries for 2,3-DHDB (yellow), NAD^+^ (light blue), and relevant amino acid side-chains (refer to main text). (*B*) Substrate–enzyme hydrogen-bonding, visualized as dotted lines (O-O lengths are in Ångstroms [Å]). Substrate mobility should be reduced by these bonds and the protruding side chains of Gln138 and Gln237 at the nonpolar side of the dihydrodiol ring (C4 through C6). Relative to *A*, the complex has been rotated horizontally about 90°. (*C*) Electrostatic potential mapped onto the solvent-accessible surface of the SDR57C complex (front view). NAD^+^ is shown in stick form (light blue). 2,3-DHDB is hidden for clarity. Potential values are given in units of kT per unit charge (k, Boltzmann’s constant; T, absolute temperature).

Whereas no experimental information is available for the geometry of 2,3-DHDB, the structure and thermodynamics of its four cis and trans stereoisomers have been studied by ab initio quantum calculations (38). These suggested that cis (2R,3S) is the most stable 2,3-DHDB isomer. Through AutoDock Vina calculations and superposition with the X-ray structure of BphB_B356_ bound to its catechol product (35), the 2R,3S isomer was docked at the putative active site of SDR57C (Fig. 5*A*). The predicted substrate-binding pocket consists of a deep cavity with two distinct regions. The outer zone would hold the benzene ring of 2,3-DHDB near the side chains of Val186, Pro193, Leu241, Leu249, and Pro231. The dihydrodiol ring would bind to the inner zone, which contains the invariant residues Tyr199 and Lys203, the nicotinamide ring of NAD^+^, and the polar residues Ser185 and Thr187 (Fig. 5*B*). The 3-OH group of 2,3-DHDB and the side chains of Tyr199 and Ser185 would be situated at hydrogen-bonding distances (O-O lengths of 2.9 and 2.5 Å, respectively). A third bond can be predicted between the γO of Thr187 and the 2-OH group of 2,3-DHDB (O-O length, 2.6 Å). Stabilizing groups have also been hypothesized for bacterial BphB enzymes despite the lack of atomic coordinates for 2,3-DHDB (35). The electrostatic potential calculated on the molecular surface (Fig. 3*C*) adds to the distinctive features of the substrate-binding cavity. There is a sharp contrast between the inner zone, in which Lys203 and other basic groups make a significant positive contribution, and the outer nonpolar zone, markedly neutral. Such asymmetry would favor the orientation proposed here for 2,3-DHDB in the ternary complex. Based on our docking and biochemical data as well as structural similarities with other SDR enzymes (25), a two-step reaction mechanism can be envisaged for SDR57C (*SI Appendix*, Fig. S11). Invariant Lys203 and Tyr199 (which are spatially conserved too; Fig. 1*D*) conform an extended proton relay system along with NAD^+^, whereby Lys203 decreases the p*K*_a_ of the hydroxyl group of Tyr199 with the assistance of NAD^+^ ribose. Tyr199 can thus act as a catalytic base that deprotonates the three-hydroxyl group of 2,3-DHDB. The neighboring nicotinamide moiety of NAD^+^ would accept the other H atom at the C3 of 2,3-DHDB along with two electrons (hydride transfer), rendering NADH and a hydroxyl ketone intermediate. In a second step, keto-enol tautomerization of the oxidized intermediate would yield 2,3-DHB, a product empirically validated here (Fig. 2*D*). Such tautomerization is probably nonenzymatic, a notion supported by the ring rearomatization occurring when 2,3-DHDB is oxidized and by the low-energy barriers calculated for its conversion to 2,3-DHB (38). The mechanism proposed here is thus cogent with 1) the detection of 2,3-DHB and HOPDA in our functional studies (Fig. 2 D–F), 2) the in silico geometry for the ternary SDR57C-NAD^+^-DHDB complex (Fig. 5 and *SI Appendix*, Fig. S11), 3) theoretical calculations (38), and 4) the general catalytic features of SDR enzymes, including the strict conservation of Tyr and Lys at the active site (25).

## Concluding Remarks

Virtually all organisms activate biochemical machinery to deal with toxic substances. Plants do so for biphenyl compounds (e.g., 14–16). However, a clear-cut catabolic pathway has not been proposed until now. After identifying SDR57 as a major PCB-responsive enzyme in poplar, our results suggest that plants catabolize biphenyl skeletons in a manner similar to aerobic bacterial degraders. It is noteworthy that the upper catabolic pathways of both taxa involve distant enzymes with remarkably similar geometries, especially at the active site. It is equally noteworthy that their encoding genes are activated by biphenyl exposure. The difference in SDR57C regulation by PCBs between *Arabidopsis* and *Populus *can be explained in part by differences in gene expression but may also be related to differences in the endogenous oxidation of natural biphenyls such as phytoalexins (39) and microbial compounds, lignin-derivatives included (40). On the applied side, our results open avenues to select superior genotypes or improve existing ones for environmental cleanup. Poplars and other short-rotation trees are excellent targets for remediating POPs (9–11), including PCBs and biphenyl. Biphenyl is an important toxicant itself that continues to be produced extensively (41).

## Materials and Methods

### Plant Material and Treatments.

*P. tremula* L. x *P. alba* L. INRA 717–1B4 and *A. thaliana* Col-0 were used throughout this study. Biphenyl (Sigma-Aldrich) or Aroclor 1221 (Scharlab) were dispersed in culture media with a hot plate magnetic stirrer. We used freshly prepared 1,000-fold concentrated solutions in DMSO, so that the DMSO concentration was identical in all culture media (0.1%, vol/vol) regardless of the toxicant concentration. Nontreated control plants were exposed to the same DMSO concentration. *Arabidopsis* seeds were germinated on agar (7 g/L) plates at pH 5.7 containing half-strength MS salts with vitamins (Duchefa M0222), 20 g/L sucrose, and 0.5 g/L 2-(4-morpholinyl)ethanesulfonic acid (MES). After 5 d, seedlings were carefully transferred to the same medium supplemented with DMSO or biphenyl (up to 300 mg/L) in DMSO. Plates were kept at 22 °C for 17 additional d under white cool light (130 µmol/m^2 ^x s) and long-day conditions. Except for the growth–dose curves (9 seedlings per plate), we routinely placed six seedlings of each line per plate. Plates were kept at 22 °C for 17 additional d under white cool light (130 µmol/m^2 ^x s) and long-day conditions. For assays in liquid medium, sterile 25 × 150 mm borosilicate glass tubes (Kimble Chase) were prepared containing 10 mL of the following medium at pH 5.7: MS salts with vitamins, 10 g/L sucrose, 0.5 g/L MES, and up to 50 mg/L of biphenyl in DMSO, freshly prepared as above. Seven seedlings germinated on solid medium for 5 d were placed per tube and kept 14 d at 22 °C under continuous shaking (200 rpm) and lighting (130 µmol/m^2^ x s). The medium was replaced once a week. For fresh biomass measurements in solid and liquid cultures, plantlets were washed with sterile water, briefly blotted with paper, and weighed. We conducted four independent replicas of all treatments, with at least 30 (solid medium) or 35 (liquid medium) plantlets being assayed per line and replica. Poplar plants were kept and grown as described (42). Freshly obtained cuttings were grown for 4 wk in shoot propagation medium and transferred to rooting medium until newly formed roots were ∼0.5 cm long (8 to 10 d). At this stage, they were carefully transferred to fresh rooting medium supplemented with Aroclor 1221 (100 mg/L) or biphenyl (150 mg/L), each dissolved in DMSO and dispersed as described above. For nontreated controls, only DMSO (0.1%, vol/vol) was added. The flasks were kept at 24 °C under white cool light (130 µmol/m^2 ^x s) and long-day conditions.

### Protein Analysis and Identification.

Frozen leaf powder was suspended at 4 mL/g in 1 M Tris⋅HCl, pH 8.0, 7 M urea, 2 M thiourea, 65 mM 3-[(3-cholamidopropyl)dimethylammonio]-1-propanesulfonate (CHAPS), 0.5% (wt/vol) Triton X-100, 100 mM dithiothreitol (DTT), and 1% (vol/vol) of a protease inhibitor mixture for plant cell and tissue extracts (Sigma-Aldrich P9599). After shaking for 60 min at 4 °C, insoluble material was removed (2 × 20 min, 16,000 g, 4 °C). Supernatant aliquots (0.1 vol) were mixed with an equal volume of 20% (wt/vol) TCA, 20 mM DTT in 80% acetone, and kept 2 h at 4 °C followed by centrifugation (20 min, 13,200 g, 4 °C). Pellets were washed twice with 20 mM DTT in 90% acetone, speed-vac dried, and suspended in 4.8 M Urea, 0.2 M Tris⋅HCl, pH 8.0. Protein content was determined with the compatible Coomassie Plus Assay Reagent from Pierce. Suitable aliquots were TCA-precipitated for further analysis. For one-dimensional sodium dodecyl sulfate-polyacrylamide gel electrophoresis (SDS-PAGE), proteins were suspended in 62.5 mM Tris⋅HCl, pH 6.8, 8 M Urea, 0.2% (wt/vol) SDS, 5% (wt/vol) 2-mercaptoethanol, and fractionated in a Bio-Rad Miniprotein II system. For isoelectric focusing (IEF) × SDS-PAGE, all specific reagents and equipment were from GE Healthcare. Proteins were suspended in 20 mM Tris, pH 8.0, 7 M urea, 2 M thiourea, 65 mM CHAPS, 100 mM DTT, and supplemented with 0.5% (vol/vol) commercial IPG buffer pH 3 to 10. Proteins (750 μg in 350 μL) were applied to 18-cm-long immobilized pH gradient strips pH 3 to 10. IEF was run in an Ettan IPGphor II IEF unit using a six-step program: 6 h at 30 V, 6 h at 60 V, 1 h at 500 V, 1 h at 1,000 V, 3 h at 4,500 V, and 8 h at 8,000 V (55,000 V and h in total). Strips were then washed with water and subjected to two incubations at room temperature: 1) 20 min in 50 mM Tris⋅HCl, pH 6.8, 6 M Urea, 2% (wt/vol) SDS, 130 mM DTT, and 30% (vol/vol) glycerol and 2) 20 min in the same solution, except that bromophenol blue (0.001%, wt/vol) was added, and DTT was replaced by 135 mM iodoacetamide. The electrofocused proteins were then subjected to SDS-PAGE in 12% acrylamide gels (2.6% C) in an Ettan Dalt-Six system. Separations were run at 3 W per gel for 30 min (20 °C) and then at 18 W per gel (15 °C) for 4.5 h. Proteins were visualized by Coomassie Brilliant Blue R250 staining. Selected proteins were subjected to in situ tryptic digestion, and the eluted peptides were characterized at the Proteomics Service of Universidad Complutense de Madrid (4800 Proteomics Analyzer from AB SCIEX). MS data searches were conducted with Mascot 2.0 (Matrix Science). For BLAST (basic local alignment search tool) searches of Mascot hits, we accessed the latest genome assemblies of *P. trichocarpa* (version 3 at Phytozome) and *P. tremula* x *P. alba* 717–1B4 (version 2 at AspenDB).

### DNA Constructs.

All primers are listed in *SI Appendix*, Table S2. Poly(A+) mRNA was obtained from 75 μg of poplar RNA using the Dynabeads mRNA Purification Kit (Thermo Fisher Scientific), and complementary DNA (cDNA) was synthesized with the PrimeScript RT Reagent Kit (Takara). The coding sequence of *P. tremula* SDR57C was PCR-amplified with primers *SacI-SDR-F* and *KpnI-SDR-R* and inserted into pBluescript SK(–) (Stratagene) opened with *Sac*I and *Kpn*I. The resulting construct was used as template to generate a bacterial expression cassette by two sequential PCR amplifications: 1) primer *attB1-SDR57C-F* was used to place upstream the start codon i) a partial *attB1* site for Gateway cloning (AAAAAGCAGGCT), ii) a bacterial ribosome-binding site, and iii) 9 nt of the 5′-untranslated region of the *SDR57C* gene from *P. trichocarpa* (AAACAAACA); primer *attB2-SDR57C-R* was used to place downstream the stop codon (i) 6 nt of the 3′-untranslated region of the same gene (TTGATC) and (ii) a partial *attB2* site (ACCCAGCTTTCTT); and 2) primers *nested-attB1-F* and *nested-attB2-R* were used to complete both *attB* sites. The final amplified fragment was recombined into Gateway pDONR221 and then into Gateway pDEST14 (Thermo Fisher Scientific). To shift selection from ampicillin to kanamycin, the *T7*-driven expression cassette in pDEST14 was PCR-amplified with primers *ApaI-SDR-F* and *SDR-EcoRV-R* and cloned into pDONR221 opened with *Apa*I and *EcoR*V to generate plasmid pGW-SDR. For control experiments, pDONR 221 opened with the same enzymes was blunted and relegated to itself to generate pGW. Two cassettes based on pGW-SDR were designed (*SI Appendix*, Figs. S7 and S8) for *E. coli* expression of specific components of the *bph* operon from P. *furukawaii* KF707. These constructs were synthesized at Biomatik (Delaware) and cloned into pBluescript SK(+) (*Kan*^R^) to express the enzyme BphB (plasmid pB) or cloned into pUC57 (*Amp*^R^) to jointly express enzymes BphA1, BphA2, BphA3, BphA4, and BphC (plasmid pAC). Competent bacterial cells were transformed with one plasmid (pGW-SDR, pB, or pAC) or two plasmids (pB/pAC or pGW-SDR/pAC) (Fig. 2). To maximize efficiency, cells harboring plasmids pGW-SDR or pB were cultured in LB-Kanamycin^50^ and competent cells were prepared before pAC introduction. Besides antibiotic selection, six colonies were randomly chosen after each transformation, and the presence of the appropriate construct(s) was confirmed via DNA miniprep.

### Bacterial Expression of SDR57C and Bph Enzymes.

*E. coli* BL21(DE3)pLysS was used as the host strain. Starter 2-mL cultures were grown in LB broth (16 h, 30 °C, 250 rpm) and then diluted 1:100 in 200-mL flasks containing 20 mL of LB broth and suitable antibiotics: 50 mg/L kanamycin (pGW, pGW-SDR, and pB), 50 mg/L ampicillin (pAC), and/or 25 mg/L chloramphenicol (pLysS maintenance). When the optical density at 600 nm (OD_600_) was ∼0.4, protein expression was induced with 0.5 mM IPTG (not added in control cultures). For functional studies, induction was held for 60 min, and cells were pelleted (10 min, 4,000 g, 10 °C), washed twice with cold 50 mM potassium phosphate, pH 7.5, and suspended in 1/20 vol of the same buffer. Suspensions were transferred to sterile glass tubes, and biphenyl was added at 100 mg/L. After shaking at 200 rpm and 30 °C for 5 h, the compounds present in the supernatants were analyzed by GC/MS. Two randomly selected colonies were analyzed for each construct set.

### qRT-PCR Analyses.

RNA extraction was different for poplar (42) and *Arabidopsis* (43) samples. The RNeasy mini kit (Qiagen) was used for RNA cleanup and the PrimeScript RT Reagent Kit (Takara) for cDNA synthesis. qRT-PCR amplifications were performed in triplicate in an Eco Real-Time PCR System (Illumina). Three biological replicates were analyzed per line. For each replica, material from four plants was pooled before RNA extraction. Each reaction contained ∼10 ng cDNA and 0.5 µM of each specific primer (*SI Appendix*, Table S2). Cycling conditions were the following: 10 min at 95 °C and 50 cycles of 15 s at 95 °C and 60 s at 60 °C, linked to a dissociation stage routine to detect nonspecific amplifications. As reference genes for qRT-PCR data analysis, we used *ACT8* or 1*8S rRNA* (*Arabidopsis*) and *18S rRNA* or *UBQ7* (*Populus*). Essentially, the same results were obtained with either reference.

### Expression of Poplar SDR57C in *Arabidopsis* Plants.

The cDNA for SDR57C cloned in pDONR221 (*DNA Constructs*) was recombined into the binary vector pB2GW7 (VIB, Belgium) using LR clonase (Thermo Fisher Scientific). pB2GW7 contains a CaMV *35S* promoter upstream the *attR1* site. The complete 5′ and 3′ untranslated sequences are shown in *SI Appendix*, Fig. S9*A*. The final construct was introduced into *A. thaliana* Col-0 using *Agrobacterium tumefaciens* strain C58C1(pMP90) and the floral dip method (44). Transformants were screened on 10 mg/L of glufosinate ammonium (Sigma-Aldrich). Ten independent transgenic lines, randomly chosen from two distinct transformations, were analyzed for accumulation of *SDR57C* transcripts (*SI Appendix*, Fig. S9*B*). Biphenyl treatments were performed on the T4 homozygous lines shown in this figure.

### GC/MS Detection of Bph/SDR57C Products.

The supernatants of biphenyl-treated whole-cell assays (1 mL) were extracted with 1 mL of ethyl acetate under acidic conditions. After solvent evaporation in vacuo, samples were methylated with (trimethylsilyl)diazomethane (Sigma-Aldrich) and evaporated again. They were then suspended in dichloromethane and injected into a Hewlett Packard 6890 gas chromatograph equipped with a selective mass detector (HP 5973) and an Agilent ATM-5 column (250 × 0.25 mm; 0.20 μm pore). Helium was used as the carrier gas at a flow rate of 0.8 mL/min. Oven temperature was raised from 60 to 300 °C (held for 20 min) at 6 °C/min, and the sample injector was kept at 275 °C. For quantitative measurements, decafluorobiphenyl was added to the extracts at 1 μg/L as an internal standard. The Wiley Registry of Mass Spectral Data were used for component identification. The distribution of 2,3-DHB was obtained from the m/z 186 chromatograms and that of HOPDA from m/z 232 chromatograms.

### Three-Dimensional Modeling and Molecular Docking.

The structural model for *P. tremula* SDR57C was generated with SWISS-MODEL (45). Alternative models were generated with the I-TASSER (iterative threading assembly refinement) server (46). Structural alignments were performed with PyMol 1.7.6.7 (Schrödinger, LLC). Geometries were optimized by energy minimization with University of California San Francisco (UCSF) Chimera 1.13.1 (47) applying Amber parameters. Proteins structurally similar to optimized SDR57C and *P. furukawaii* BphC were searched for with mTM-align (48) and PDBeFold version 2.59 (49). Both engines were selected for not rejecting dissimilar sequences. NAD^+^ docking simulations were based on AutoDock Vina calculations (50) and the X-ray structures of BphB_LB400_ (PDB code 1BDB, chain A) and a putative SDR from *B. cenocepacia* bound to NAD^+^ (PDB entry 5JYD). Substrate docking was based on the X-ray coordinates of 2,3-DHB bound to BphB_B356_ (PDB code 3ZV5, chain A). Considering substrate proximity, SDR57C residues 134 through 142, 185 through 204, 225 through 241, and 248 through 256 were not fixed during energy minimizations (RMSD for the best pose: 0.13 Å; 830 atoms updated). The electrostatic Poisson–Boltzmann (PB) potential was deduced with the APBS (adaptive Poisson-Boltzmann solver) tools in PyMol, assigning AMBER atomic charges and radii. The nonlinear PB equation was solved in sequential multigrid calculations at 298.15 K, with dielectric constants of 2 for proteins and 78.54 for water. Mapping of PB potentials onto protein surfaces was rendered with PyMOL. Other molecular graphics were prepared with UCSF Chimera.

### Phylogenetic Analyses.

Amino acid sequences for SDR57C from *P. tremula*, *P. alba*, and *P. trichocarpa* (XP_002311602) were used to search the *P. trichocarpa* genome (version 3) for similar sequences (>25% identity and >85% coverage) using the BLAST tool TBLASTN at Phytozome. Only one match was considered for families with nearly identical members (>85% identity). Phylogenetic trees were built and visualized as described in *SI Appendix*, Fig. S2.

### Data Analysis.

Statistical analyses were performed with Stata version 16 (StataCorp). Normality was assessed with the Anderson–Darling test (*P* ≤ 0.05), based on the empirical distribution function. One-sided Student's *t* tests were used to calculate significant differences. For IC_50_ estimates, dose–response data were fitted to a four-parameter logistic (sigmoidal) function using Stata. Setting the minimal response to zero produced nearly identical results.

## Supplementary Material

Supplementary File

## Data Availability

All study data are included in the article and/or *SI Appendix*.
