# Supplementary material for: A poplar short-chain dehydrogenase reductase plays a potential key role in biphenyl detoxification
Source: Proc Natl Acad Sci U S A. 2021 Aug 26;118(35):e2103378118. doi: 10.1073/pnas.2103378118 (PMC8536390; doi:10.1073/pnas.2103378118)
Supplement: Supplementary File [file pnas.2103378118.sapp.pdf]

PNAS
www.pnas.org

Supplementary Information for

A poplar short-chain dehydrogenase reductase plays a potential key role in biphenyl detoxification.

Ángela Contreras, Irene Merino, Enrique Álvarez, David Bolonio, Jose-Eugenio Ortiz, Luis Oñate-Sánchez, Luis Gómez

Corresponding author: Luis Gómez
Email: luis.gomez@upm.es

This PDF file includes:

Figures S1 to S11
Tables S1 to S2
SI References

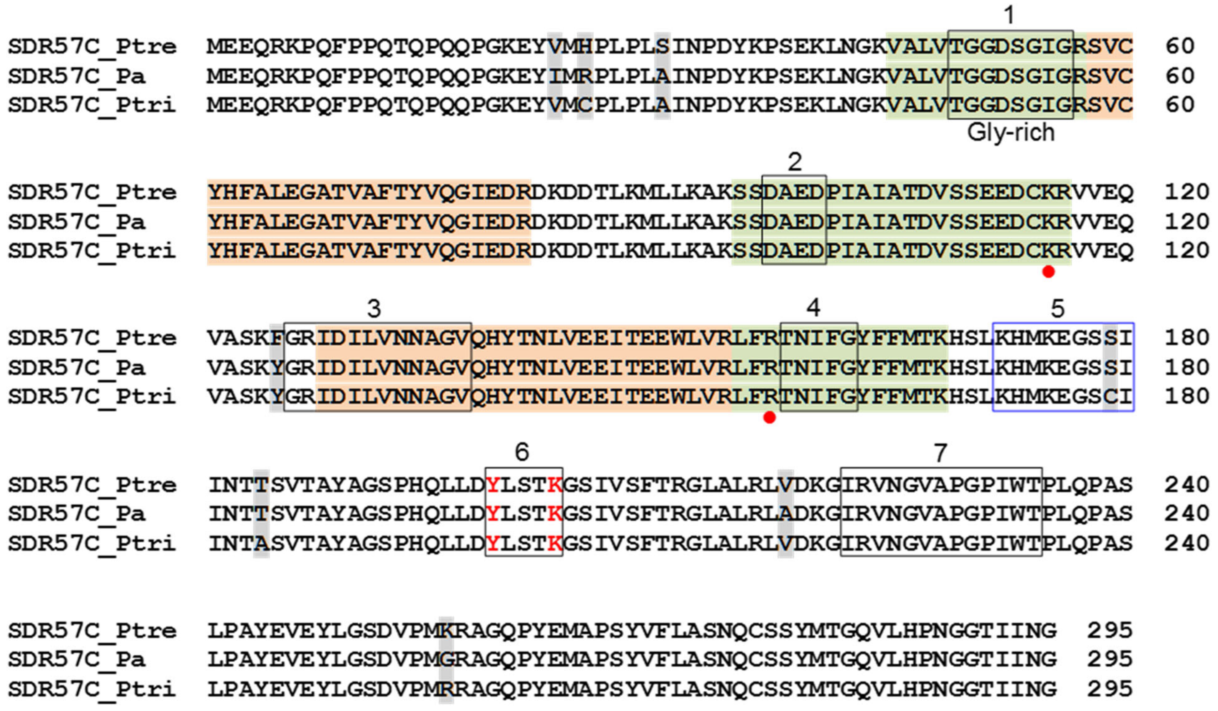

Figure S1. Sequence alignment of the single SDR57C component encoded in the genomes of *P. tremula*, *P. alba* and *P. trichocarpa* (gene model POPTR_008G149200v3 at NCBI). Seven characteristic motifs (1) are boxed. Green and pink shading, peptides identified by mass-spectrometry (*SI Appendix*, Table S1); a truncated form of peptide 147-160 (LFR…MTK) lacking the first three residues was also detected. Grey shading, non-identical positions. Red dots, missed trypsin cuts. The putative catalytic residues are highlighted in red (box 6).

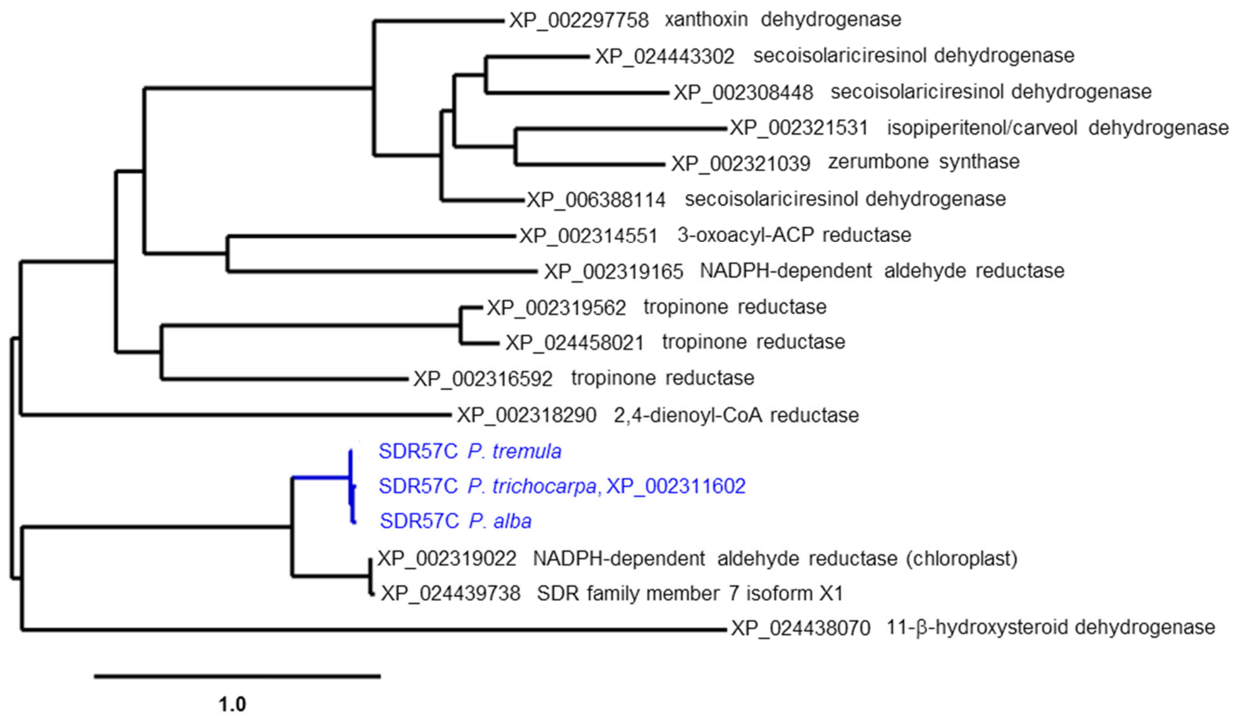

Figure S2. Phylogram of SDR57C and related SDR proteins. It includes the SDR57C members of *P. tremula, P. alba and P. trichocarpa* (blue color) along with the closest proteins encoded by the *P. trichocarpa* genome (see main text). All have been classified as SDRs. Only one member was considered for subfamilies with highly similar members (> 85% identity). For tree building and visualization we used PhyML and TreeDyn as described (4), except that protein sequences were aligned with ClustalW (EMBL-EBI). Branches with bootstrap values less than 50% were collapsed (500 replicate trees).

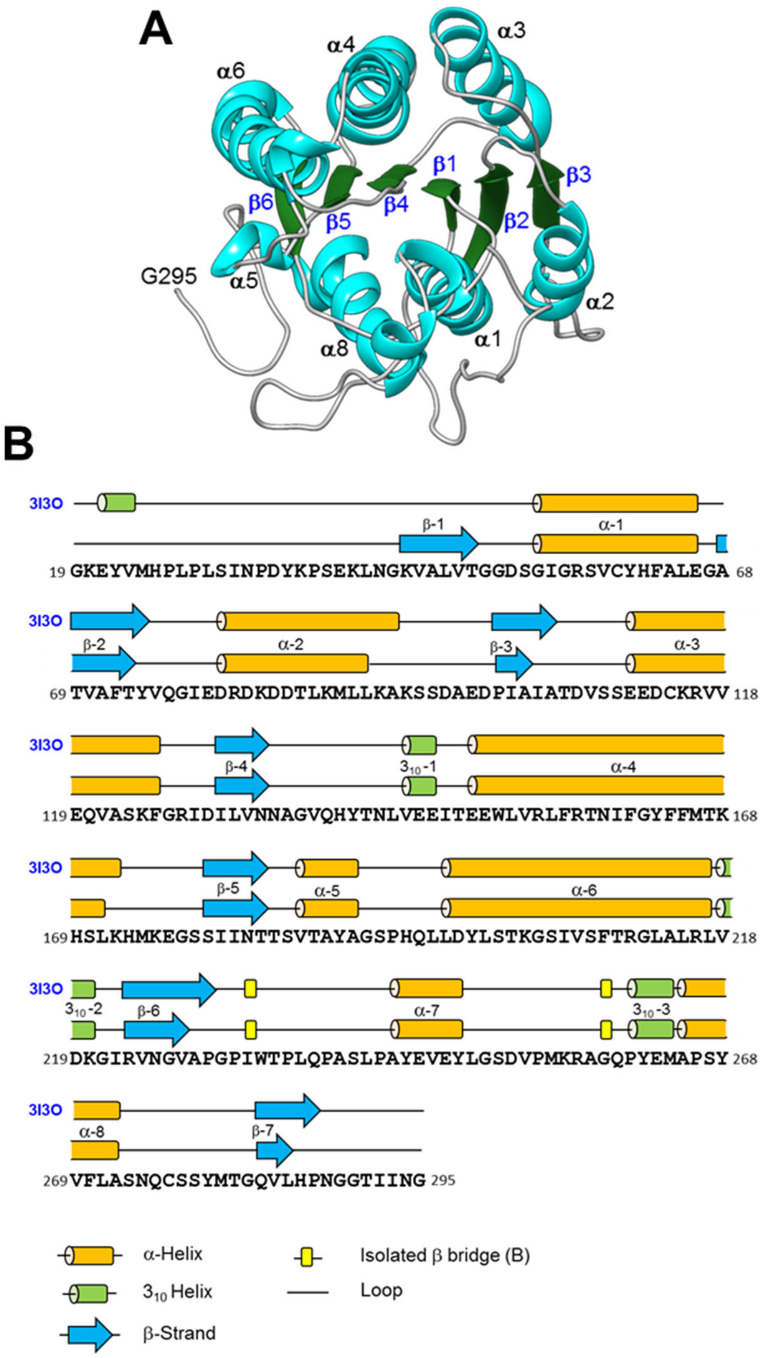

Figure S3. Model structure features for *P. tremula* SDR57C. (*A*) Apoenzyme overall fold predicted by SWISS-MODEL (residues 19-295) and colored according to secondary structure: α-helices, blue; β-sheets, green. Virtually identical models were predicted for the equivalent proteins of *P. alba* and *P. trichocarpa* (RMSD < 0.2 Å for all binary comparisons; 277 Cα pairs compared). The model was very close to the highest-scoring fold generated by I-TASSER (RMSD of 0.53 Å; 259 Cα pairs), even though it used a different top template, namely a putative SDR from *Burkholderia cenocepacia* bound to NAD^+ (PDB code 5JYD; 42% identity). (*B*) The secondary structure assigned to SDR57C with STRIDE (5) is compared with X-ray information for its top-ranked template in SWISS MODEL: a SDR from *Bacillus anthracis* strain 'Ames Ancestor' crystallized in complex with NAD^+-acetone (PDB code: 3I3O). Essentially the same secondary structure was predicted for SDR57C by SWISS-MODEL.

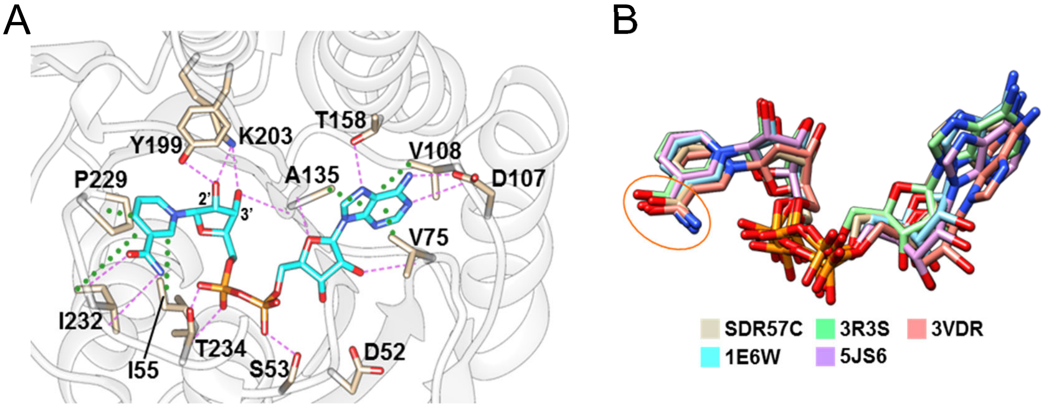

Figure S4. Modelled docking of NAD⁺ to *P. tremula* SDR57C. (*A*) The putative coenzyme binding site is a cavity curved around the long coil between strand 4 and helix 4. Dashed magenta lines represent hydrogen bonds, which involve amino acid side chains and main chain atoms. Hydrophobic interactions with the NAD⁺ rings are visualized by green dotted lines. The nicotinamide ring is sandwiched between Pro229, Ile55, Ile232, and Leu236 (the latter is hidden for clarity). The side chains of Val75, Val108, and Ala135 would help position the adenine moiety. (*B*) Spatial position of NAD⁺ or NADH after structural alignment of SDR57C-NAD⁺ with the X-ray structures of the following SDR enzymes: *Salmonella enterica* YghA oxidoreductase (PDB code: 3R3S; coenzyme: NAD⁺), rat brain 3-hydroxyacyl-CoA dehydrogenase (1E6W; NADH), *Homo sapiens* 17 β-hydroxysteroid dehydrogenase (5JS6; NAD⁺), and *Alcaligenes faecalis* D-3-hydroxybutyrate dehydrogenase (3VDR; NAD⁺). These empirical structures were not used as modelling templates. The alignment was based solely on apoenzyme geometries (hidden for clarity). NAD⁺ coloring: N, blue; O, red; P, yellow. Amide groups are encircled.

```
SDR57C   MEEQRKPQFPPQTQPQQPGKEYVMHPLPLSINPDYKPSEKLNGKVALVTGGDSGIGRSVC   60
BphB                                           MKLKGEAVLITGGASGLGRALV   22

SDR57C   YHFALEGATVAFTYVQGIEDRDKDDTLKMLLKAKSSDAEDPIAIATDVSSEEDCKRVVEQ  120
BphB     DRFVAEGAKVAVL----------DKSAERLAELETDHGDNVLGIVGDVRSLEDQKQAASR   72

SDR57C   VASKFGRIDILVNNAGVQHYTNLVEEITEEWLV----RLFRTNIFGYFFMTKHSL--KHM  174
BphB     CVARFGKIDTLIPNAGIWDYSTALVDLPEESLDAAFDEVFHINVKGYIHAVKACLPALVA  132

SDR57C   KEGSSIINTTSVTAYAGSPHQLLDYLSTKGSIVSFTRGLALRLVDKGIRVNGVAPGPIWT  234
BphB     SRGNVIFTISNAGFYPNGGGPL--YTAAKHAIVGLVRELAFELAPY-VRVNGVGSGGINS  189

SDR57C   PLQ-PASLPAYE--------VEYLGSDVPMKRAGQPYEMAPSYVFLASNQCSSYMTGQVL  285
BphB     DLRGPSSLGMGSKAISTVPLADMLKSVLPIGRMPEVEEYTGAYVFFATRGDAAPATGALL  249

SDR57C   HPNGGTIING                                                    295
BphB     NYDGGLGVRGFFSGAGGNDLLEQLNIHP                                  277
```

Figure S5. Amino acid sequence alignment between *P. tremula* SDR57C and *B. xenovorans* $BphB_{LB400}$ (PDB code: 1BDB). Blue shading, proposed catalytic residues (6). Black shading, invariant residues.

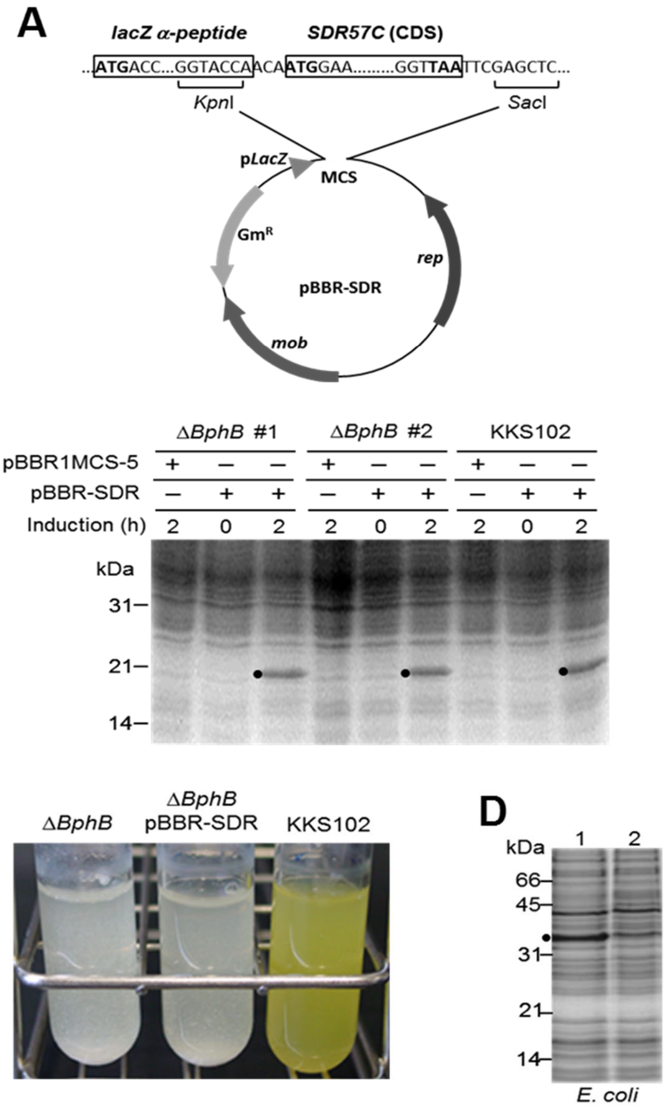

Figure S6. Expression of poplar SDR57C in *Acidovorax* sp. KKS102. (*A*) Cloning strategy. cDNA was obtained from PCB-treated poplars and the coding sequence for SDR57C was amplified with primers *KpnI-SDR-F* and *SacI-SDR-R* (*SI Appendix*, Table S2). The resulting fragment was cloned into pBBR1MCS-5 (7) to generate pBBR-SDR for expression in *Acidovorax* (*lacZ* promoter). The recombinant protein was N-terminally fused to residues 1-21 of the LacZ-alpha peptide. (*B*) SDS-PAGE fractionation of soluble proteins from the Δ*bphB* mutant and its parental strain (kindly provided by Dr. Y. Ohtsubo, Tohoku University, Japan), harboring the indicated plasmids. Cultures were grown as described (8) and expression was induced at an OD_{595} of 0.5 with 1mM isopropyl-β-D-thiogalactopyranoside. Protein patterns were analyzed by SDS-PAGE at different times. Compared with control cultures, a new protein band was detected in cultures harboring pBBR-SDR, but its apparent size suggested proteolytic processing (expected size: *ca*. 35 kDa). Two independent Δ*bphB* transformants are shown. (*D*) HOPDA, the yellow meta-cleavage product of BphC action, was visualized in KKS102 cultures (*right*) but not in Δ*bphB* cultures (*left*). Transformation of the Δ*bphB* mutant with pBBR-SDR did not result in functional complementation (*center*; up to 2 mM IPTG added), consistent with proteolysis. (*D*) A protein band with the expected size was detected by SDS-PAGE in *E. coli* DH5α cells transformed with pBBR-SDR (lane 1) but not with pBBR1MCS-5 (lane 2, control).

TCCCGCGAAATTAATACGACTCACTATAGGGAGACCACAACGGTTTCCCTCTAGAAATAATTTTGTTTAACTTTAAGAAGGAGATATACAT**ATG**
AGCTCATCAATCAAAGAAGTGCAGGGAGCCCCTGTGAAGTGGGTTACCAATTGGACGCCGGAGGCGATCCGGGGGTTGGTCGATCAGGAAAAAG
GGCTGCTTGATCCACGCATCTACGCCGATCAGAGTCTTTATGAGCTGGAGCTTGAGCGGGTTTTTGGTCGCTCTTGGCTGTTACTTGGGCACGA
GAGTCATGTGCCTGAAACCGGGGACTTCCTGGCCACTTACATGGGCGAAGATCCGGTGGTTATGGTGCGACAGAAAGACAAGAGCATCAAGGTG
TTCCTGAACCAGTGCCGGCACCGCGGCATGCGTATCTGCCGCTCGGACGCCGGCAACGCCAAGGCTTTCACCTGCAGCTATCACGGCTGGGCCT
ACGACATCGCCGGCAAGCTGGTGAACGTGCCGTTCGAGAAGGAAGCCTTTTGCGACAAGAAAGAAGGCGACTGCGGCTTTGACAAGGCCGAATG
GGGCCCGCTCCAGGCACGCGTGGCAACCTACAAGGGCCTGGTCTTTGCCAACTGGGATGTGCAGGCGCCAGACCTGGAGACCTACCTCGGTGAC
GCCCGCCCCTATATGGACGTCATGCTGGATCGCACGCCGGCCGGGACTGTGGCCATCGGCGGCATGCAGAAGTGGGTGATTCCGTGCAACTGGA
AGTTTGCCGCCGAGCAGTTCTGCAGTGACATGTACCACGCCGGCACCATGTCGCACCTGTCCGGCATCCTGGCGGGCATGCCGCCGGAAATGGA
CCTGTCGCATGCACAGGTGCCCACCAAGGGCAACCAGTTCCGGGCCGGCTGGGGCGGGCACGGCTCGGGCTGGTTCGTCGACGAGCCGGGCATG
CTCATGGCGGTGATGGGGCCCAAGGTCACCCAGTACTGGACCGAAGGTCCGGCTGCCGACCTGGCAGAACAGCGACTGGGCCACACCATGCCGG
TTCGACGCATGTTCGGCCAGCACATGAGCGTCTTCCCGACCTGCTCGTTCCTCCCGGCCATCAACACCATCCGGACCTGGCACCCGCGCGGCCC
CAACGAAATCGAAGTGTGGGCCTTCACCTTGGTCGATGCCGATGCCCCGGCCGAGATCAAGGAAGAATATCGCCGGCACAACATCCGCACCTTC
TCCGCAGGCGGCGTGTTTGAGCAGGACGATGGCGAGAACTGGGTGGAGATCCAGAAGGGGCTACGTGGGTACAAGGCCAAGAGCCAGCCGCTCA
ATGCCCAGATGGGCCTGGGTCGGTCGCAGACCGGTCACCCTGATTTTCCTGGCAACGTCGGCTACGTCTACGCCGAAGAAGCGGCGCGGGGTAT
GTATCACCACTGGATGCGCATGATGTCCGAGCCCAGCTGGGCCACGCTCAAGCCCTGATCAAGACGCAATCGTTAGATCTGTCAACCGGAAGAA
TTCAAC**ATG**GTGGGCTGGACGTGCATGTGCAGACGGCGCGCCGAGGTTCCGTCCCCTGATATTTACTTGGAGATAACTGTTATGACAAATCCAT
CCCCGCATTTTTTCAAAACATTTGAATGGCCAAGCAAGGCGGCTGGCCTTGAGTTGCAGAACGAGATCGAGCAGTTCTACTACCGCGAAGCGCA
GTTGCTTGACCACCGGGCCTACGAGGCCTGGTTTGCCCTGCTGGACAAAGATATCCACTACTTCATGCCGCTGCGCACCAATCGCATGATCCGG
GAGGGCGAGCTGGAATATTCCGGCGACCAGGATGTTGCCCATTTCGATGAAACCCATGAAACCATGTACGGGCGCATCCGCAAGGTGACCTCGG
ACGTGGGCTGGGCGGAGAACCCGCCTTCCCGCACGCGCCACCTGGTCTCCAACGTCATCGTCAAGGAGACGGCCACGCCGGATACCTTCGAGGT
CAATTCCGCATTCATCCTGTACCGCAATCGGCTTGAGCGCCAGGTCGACATCTTCGCGGGCGAACGCCGGGACGTGCTGCGCCGCGCCGACAAC
AACCTTGGTTTCAGCATCGCCAAGCGCACCATCCTGCTCGACGCCAGTACCTTGCTGTCGAACAACCTGAGCATGTTCTTCTAGATTACTCTTC
AGCCACCAACAGTGACTGTTCGCCCCAGGCGATTTAACCCTTTTAACTAATTACAAGAAGCGTT**ATG**AAATTTACCAGAGTTTGTGATCGAAGA
GATGTGCCCGAAGGCGAAGCCCTGAAGGTCGAAAGTGGAGGCACCTCCGTCGCGATTTTCAATGTGGATGGCGAGCTGTTCGCAACACAGGACC
GCTGCACCCACGGCGACTGGTCCCTGTCCGATGGCGGCTATCTTGAAGGTGACGTGGTGGAATGCTCACTGCACATGGGGAAGTTTTGCGTTCG
CACGGGCAAGGTCAAATCACCGCCGCCCTGTGAGGCACTGAAGATATTTCCGATCCGCATCGAAGACAATGACGTGCTGGTCGACTTCGAAGCC
GGGTATCTGGCGCC**ATG**ATCGACACCATCGCCATCATCGGCGCCGGCCTGGCCGGTTCGACGGCTGCGCGCGCACTGCGCGCCCAGGGATACGA
GGGGCGCATCCACCTGCTCGGGGATGAGTCGCATCAGGCCTATGACCGGACCACGCTGTCCAAGACGGTGCTGGCGGGCGAGCAGCCCGAGCCG
CCTGCAATCCTGGACAGCGCCTGGTACGCATCGGCCCATGTGGATGTCCAGCTCGGGCGACGGGTGAGTTGCCTGGATCTGGCCAACCGCCAGA
TTCAGTTTGAATCGGGCGCCCCGCTGGCCTACGACCGGCTGCTGCTGGCCACCGGCGCGCGCGCCCGGCGCATGGCGATTCGGGGTGGCGACCT
GGCAGGCATCCATACCTTGCGAGACCTCGCCGACAGCCAGGCGCTGCGGCAGGCGCTGCAACCGGGCCAGTCGCTGGTCATCGTCGGCGGAGGC
CTGATCGGTTGCGAGGTGGCGACCACCGCCCGCAAGCTGAGTGTCCATGTCACGATTCTGGAAGCCGGCGACGAGTTGCTGGTGCGCGTGCTGG
GTCACCGGACCGGGGCATGGTGTCGGGCCGAACTGGAACGCATGGGTGTCCGCGTGGAGCGCAATGCACAGGCCGCGCGCTTCGAAGGCCAGGG
GCAGGTGCGCGCCGTGATCTGCGCCGACGGGCGCCGGGTGCCCGCCGATGTGGTCTTGGTCAGCATTGGCGCCGAGCCGGCGGACGAGCTGGCC
CGTGCCGCTGGCATCGCCTGCGCGCGCGGCGTGCTGGTCGACGCCACCGGCGCCACCTCGTGTCCAGAGGTGTTCGCCGCCGGTGACGTCGCCG
CCTGGCCGCTGCGTCAAGGGGGCCAGCGCTCGCTGGAGACCTACCTGAACAGCCAGATGGAGGCCGAAATCGCGGCCAGCGCCATGTTGAGTCA
GCCCGTGCCGGCGCCCCAGGTGCCGACCTCGTGGACGGAGATTGCAGGCCACCGCATCCAGATGATTGGCGATGCCGAAGGGCCCGGCGAGATC
GTCGTACGCGGCGACGCCCAGAGCGGCCAGCCAATCGTGTTGCTCAGGCTGCTTGATGGCTGCGTCGAGGCCGCGACGGCGATCAATGCCACCA
GGGAATTTTCTGTGGCGACCCGACTGGTCGGCACCCGGGTTTCTGTTTCCGCCGAGCAACTGCAGGACGTCGGCTCGAACCTGCGGGATTTACT
CAAAGCCAAACCGAATTGAGCATCGACGAAGGAGACAGTA**ATG**AGCATCAGAAGTTTGGGATACATGGGGTTTGCGGTCAGCGACGTAGCTGCT
TGGCGTTCGTTTCTGACGCAGAAACTGGGCTTGATGGAAGCGGGCACGACCGACAACGGCGACCTGTTCCGCATCGATTCGAGAGCCTGGCGGA
TCGCCGTTCAGCAGGGCGAGGTTGACGATCTGGCCTTTGCCGGCTACGAGGTGGCCGATGCGGCAGGGCTGGCGCAGATGGCTGACAAGCTCAA
ACAGGCCGGTATCGCAGTCACCACCGGCGATGCCTCATTGGCCAGGCGCCGCGGGGTGACCGGATTGATCACCTTTGCCGACCCGTTTGGCCTG
CCGTTGGAAATTTACTATGGCGCCAGCGAGGTGTTCGAAAAACCGTTCCTGCCTGGTGCGGCCGTGTCGGGTTTCCTGACCGGCGAGCAAGGGC
TGGGGCATTTCGTGCGCTGCGTTCCGGATTCGGACAAGGCGCTGGCGTTTTATACCGACGTGCTCGGCTTCCAGTTGTCTGACGTCATCGACAT
GAAAATGGGGCCGGACGTGACGGTTCCTGTGTACTTCCTGCACTGCAACGAACGCCACCACACCCTGGCAATTGCGGCATTCCCGCTGCCAAAA
CGCATTCATCACTTCATGCTCGAAGTCGCCTCGCTCGATGACGTCGGCTTTGCATTTGATCGGGTTGACGCGGACGGCTTGATCACCTCCACGC
TGGGGCGCCACACCAATGACCACATGGTGTCGTTCTATGCCTCGACCCCGTCCGGAGTAGAGGTCGAGTATGGCTGGAGTGCCCGTACCGTTGA
CCGCTCCTGGGTTGTGGTGAGGCACGACAGTCCGAGCATGTGGGGCCACAAGTCTGTGCGCGACAAAGCAGCTGCGCGCAACAAAGCATGAACA
ACAACCTGAATAGCACGAACCCCTTGGGGCCTCTAAACGGGTCTTGAGGGGTTTTTTG

Figure S7. Nucleotide sequence synthesized and cloned into the *EcoR*V site of pUC57 (Amp^R) to render plasmid pAC. It contains five structural genes from the *bph* operon of *P. furukawaii* KF707 (ENA sequence: M83673.1, assembly GCA_000262065): (i) the terminal dioxygenase large subunit (*bphA*1); (ii) the terminal dioxygenase small subunit (*bphA*2); (iii) ferredoxin (*bphA*3); (iv) NADH-dependent ferredoxin reductase (*bphA*4); and (v) 2,3-dihydroxybiphenyl dioxygenase (*bph*C). The original 4 bp-overlap between *bphA*3 and *bphA*4 was kept. The 5' and 3' terminal regions were based, respectively, on pGW-SDR (see main text and Fig. 2*A*) and the 3'-untranslated region (20 bp) of *bph*C, followed by the terminator region of plasmid pET30a (Novagen). Highlighted elements: *T7* promoter (green), RBS (yellow), coding sequences (grey), start codons (bold), stop codons (underlined), and pET30a terminator (cyan).

ATCCCGCGAAATTAATACGACTCACTATAGGGAGACCACAACGGTTTCCCTCTAGATCACAAGTTTGTACAAAAAAGCAGGCTAGAAGGAGTTG
GAAAA**ATG**AAACTGAAAGGTGAAGCGGTACTGATCACGGGGGGCGCCTCCGGATTAGGGCGCGCGCTCGTGGACCGATTCGTGGCCGAAGGCGC
CAAAGTGGCGGTGCTCGACAAGTCGGCAGAGCGCCTTGCAGAGCTGGAAACCGATCATGGCGACAACGTGCTCGGCATCGTCGGCGATGTGCGT
TCACTGGAAGACCAGAAACAGGCTGCCAGCCGCTGCGTGGCCAGGTTCGGGAAAATCGACACCTTGATTCCCAATGCAGGCATCTGGGATTACT
CGACGGCCTTGGTCGACCTGCCCGAAGAGAGCCTCGATGCCGCGTTCGATGAGGTCTTTCACATCAATGTCAAGGGTTATATCCATGCAGTGAA
GGCCTGCCTGCCGGCCCTGGTCGCCAGCCGTGGCAACGTGATCTTCACGATCTCCAATGCGGGCTTCTATCCCAATGGCGGCGGCCCTCTTTAC
ACCGCAGCCAAGCACGCCATCGTGGGCCTGGTGCGTGAACTGGCGTTCGAGCTGGCGCCATACGTGCGCGTCAACGGCGTGGGCCGGGCGGCA
TCAATAGCGATATGAGAGGTCCTTCCTCGCTGGGAATGGGCAGCAAGGCGATTTCGACCGTACCGCTGGCCGACATGCTGAAGTCGGTGCTGCC
GATTGGTCGCATGCCCGAGGTCGAAGAGTACACCGGGGCCTATGTGTTTTTTGCCACCCGAGGCGATGCGGCGCCTGCGACCGGGGCCTTGCTG
AACTACGACGGTGGCTTGGGCGTTCGTGGATTCTTCTCGGGTGCCGGAGGTAATGACTTGCTCGAACAGCTGAATATCCATCCCTAAGCATCGA
CGACTAGCATAACCCCTTGGGGCCTCTAAACGGGTCTTGAGGGGTTTTTTG

Figure S8. Nucleotide sequence synthesized and cloned into the *Sma*I site of pBSK(+) (*Kan*^{R}) to render plasmid pB. It contains the structural gene *bphB* from *P. furukawaii* KF707 (ENA sequence: M83673.1). Except the 9 bp upstream the start codon and the 10 bp downstream the stop codon, which are from the original KF707 *bph* operon, the 5'- and 3'-untranslated regions are based on pGW-SDR (Fig. 2*A*). The T7 promoter, RBS, and intervening region are therefore identical in pB and pGW-SDR. Highlighted elements: *T7* promoter (green), RBS (yellow), coding sequence (grey), start codon (boldface), stop codon (underlined), and terminator region of pGW-SDR (cyan).

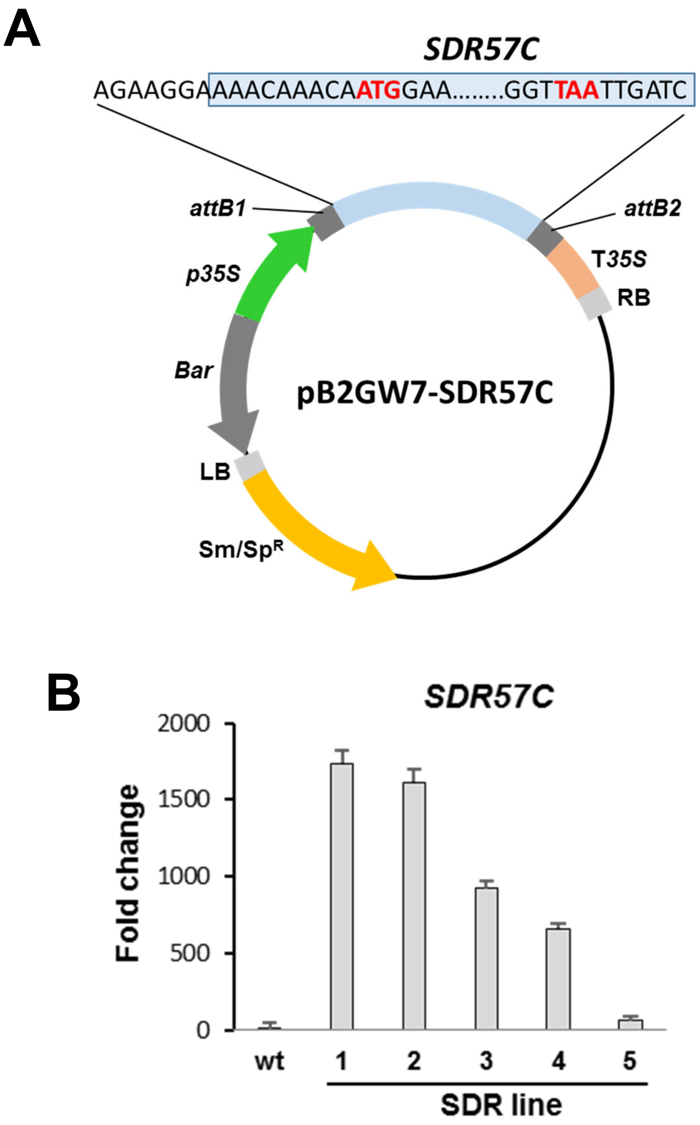

Figure S9. Overexpression of *P. tremula* SDR57C in *A. thaliana.* (*A*) Total leaf RNA was obtained from PCB-treated poplar plants and the coding sequence for SDR57C was amplified from cDNA using primers *attB1-SDR57C-F* and *attB2-SDR57C-R*. The att*B* sites were completed with primers *nested-attB1-F* and *nested-attB2-R*, and the resulting fragment was recombined into Gateway pDONR221 (BP cloning) and then into the Gateway binary vector pB2GW7 for plant transformation (LR cloning). The insert in pB2GW7 contains a bacterial ribosome binding site (unboxed) before the *SDR57C* sequence (boxed; the start and stop codons are highlighted). These primers are listed in *SI Appendix*, Table S2. (*B*) pB2GW7-SDR57C was introduced into *A. thaliana* Col-0 using *Agrobacterium tumefaciens* strain C58C1(pMP90) and the floral-dip method. The relative abundance of *SDR57C* transcripts was determined by qRT-PCR in leaves of ten 2-week-old transgenic lines from two independent transformations (T4). Shown are 5 lines, covering from the highest (nr. 1) to the lowest (nr. 5) observed expression. SDR lines with poor transgene expression performed similarly to wt plants when challenged with biphenyl. *ACT8* was used for data normalization. The mean value recorded in wt samples was used to index expression. Bars represent SEMs from 3 independent replicas (4 pooled plants per replica).

```
AT3G05600_AtEH1    --------------------------------------------------MEGIDHRMVS   10
AT2G26740_AtSEH    -----------------------------------------------------MEHRKVR    7
AT4G15960_abHSP1   MFFLCRKLSLSRFRHHFPLRLRRFLGENPNPTTHFSTLPDNQTKRPEKSRLDGVEHKTLK   60
AT2G26750_abHSP2   -----------------------------------------------------MEHRNVR    7
AT4G15955_abHSP3   -------------------------------------------------MDLTFDHSFVK   11
AT4G02340_abHSP4   --------------------------------------------------MEKIEHTTIS   10

AT3G05600_AtEH1    VNGITMHIAEKGP-------KEGPVVLLLHGFPDLWYTWRHQISGLSSLGYRAVAPDLRG   63
AT2G26740_AtSEH    GNGIDIHVAIQGP-------SDGPIVLLLHGFPELWYSWRHQIPGLAARGYRAVAPDLRG   60
AT4G15960_abHSP1   VNGINMHVAEKP----GSGSGEDPIILFLHGFPELWYTWRHQMVALSSLGYRTIAPDLRG  116
AT2G26750_abHSP2   GNGIDIHVAIQGP-------SDGTIVLLLHGFPELWYSWRHQISGLAARGYRAVAPDLRG   60
AT4G15955_abHSP3   VNGITMHVAEKSPSVAGNGAIRPPVILFLHGFPELWYTWRHQMVALSSLGYRTIAPDLRG   71
AT4G02340_abHSP4   TNGINMHVASIG---------SGPVILFVHGFPDLWYSWRHQLVSFAALGYRAIAPDLRG   61

AT3G05600_AtEH1    YGDSDSPESFSEYTCLNVVGDLVALLDSVAG-NQEKVFLVGHDWGAIIGWFLCLFRPEKI  121
AT2G26740_AtSEH    YGDSDAPAEISSYTCFNIVGDLIAVISALTASEDEKVFVVGHDWGALIAWYLCLFRPDRV  120
AT4G15960_abHSP1   YGDTEAPEKVEDYTYLNVDGDVVALIDAVTG-GDKAVSVVGHDWGAMIAWQLCQYRPEKV  175
AT2G26750_abHSP2   YGDSDAPAEISSFTCFNIVGDLVAVISTLI-KEDKKVFVVGHDWGALIAWYLCLFRPDKV  119
AT4G15955_abHSP3   YGDTDAPESVDAYTSLHVVGDLIGLIDAVVG-DREKVFVVGHDWGAIIAWHLCLFRPDRV  130
AT4G02340_abHSP4   YGDSDAPPSRESYTILHIVGDLVGLLDSLG---VDRVFLVGHDWGAIVAWWLCMIRPDRV  118

AT3G05600_AtEH1    NGFVCLSVPY--RSRNPKVKPVQGFKAVFGDDYYICRFQEPGKIEGEIASADPRIFLRNL  180
AT2G26740_AtSEH    KALVNLSVPFSFRPTDPSVKPVDRMRAFYGDDYYICRFQEFGDVEAEIAEVGTERVMKRL  180
AT4G15960_abHSP1   KALVNMSVLF--SPRNPVRVPVPTLRHVFGDDYYVCRFQKAGEIETEFKKLGTENVLKEF  233
AT2G26750_abHSP2   KALVNLSVPLSFWPTDPSVKPVDRMRAVYGNDYYVCRFQEVGDIEAEIAEVGTERVMKRL  179
AT4G15955_abHSP3   KALVNMSVVF--DPWNPKRKPTSTFKAFYGDDYYICRFQLLEILIKIHV-----------  177
AT4G02340_abHSP4   NALVNTSVVF--NPRNPSVKPVDAFRALFGDDYYICRFQEPGEIEEDFAQVDTKKLITRF  176

AT3G05600_AtEH1    FTGRTLGPPILPKDNPFGEKPNPNSENIELPEWFSKKDLDFYVSKFEKAGFTGGLNYYRA  240
AT2G26740_AtSEH    LTYRTPGPVIIPKDKSFWGSK---GETIPLPSWLTEEDVAYFVSKFEEKGFSGPVNYYRN  237
AT4G15960_abHSP1   LTYKTPGPLNLPKDKYFKRSE---NAASALPLWLTQEDLDYYVTKYENKGFTGPINYYRN  290
AT2G26750_abHSP2   LTYRTPGPLIIPKDKSFWGSK---GETIPLPSWLTEEDVAYFVSKFKEKGFCGPVNYYRN  236
AT4G15955_abHSP3   --------------CIVGKRY---DDSVSLPSWLTDSDVKYYVSKYEKNGFTGPVNYYRN  220
AT4G02340_abHSP4   FTSRNPRPPCIPKSVGFRGLPDPP----SLPAWLTEQDVRFYGDKFSQKGFTGGLNYYRA  232

AT3G05600_AtEH1    MDLNWELTAPWTGAKIQVPVKFMTGDFDMVYTTPGMKEYIHGGGFAADVPTLQEIVVIED  300
AT2G26740_AtSEH    FNRNNELLGPWVGSKIQVPTKFVIGELDLVYYMPGVKEYIHGPQFKEDVPLLEEPVVMEG  297
AT4G15960_abHSP1   IDRNWELTAPWTGAKIRVPVKFIIGDQDLTYNFPGAKEYINGGGFKRDVPLLDETVVLKG  350
AT2G26750_abHSP2   FNRNNELLGPWVGSKIQVPTKFVIGELDLVYYMPGVKEYIHGPQFKEDVPLIEEPVVMEG  296
AT4G15955_abHSP3   MDRTWELMGSLSNAKVKVPVKFIIGDQDLTYHIPGSKKYIHDGRFKSHVPLLDEVVVIKG  280
AT4G02340_abHSP4   LNLSWELTAPWTGLQIKVPVKFIVGDLDITYNIPGTKEYIHEGGLKKHVPFLQEVVVMEG  292

AT3G05600_AtEH1    AGHFVNQEKPQEVTAHINDFFTKLRDNNKSF-   331
AT2G26740_AtSEH    VAHFINQEKPQEILQIILDFISKF--------   321
AT4G15960_abHSP1   LGHFLHEENPDVINQHIHNFFHKFL-------   375
AT2G26750_abHSP2   VAHFLNQEKPQEILQIILDFISTF--------   320
AT4G15955_abHSP3   VGHFIHEERPDEISKHIHDYFLTF--------   304
AT4G02340_abHSP4   VGHFLHQEKPDEVTDHIYGFFKKFRTRETASL   324
```

Figure S10. Amino acid sequence alignment of *A. thaliana* epoxide hydrolases. Sequences were retrieved from TAIR10 with BLASTP, using as query the sequence of epoxide hydrolase 1 (AtEH1, AT3G05600), the best characterized member of this protein family. No significant hits were found other than those aligned. TAIR abbreviations: abHSP, alpha/beta-hydrolases superfamily protein; SHE, soluble epoxide hydrolase (AT2G26740). Shading was rendered with Boxshade 3.2 (Embnet) using a 70% threshold (black, identical residues; grey, similar residues). The alignment was performed with ClustalW.

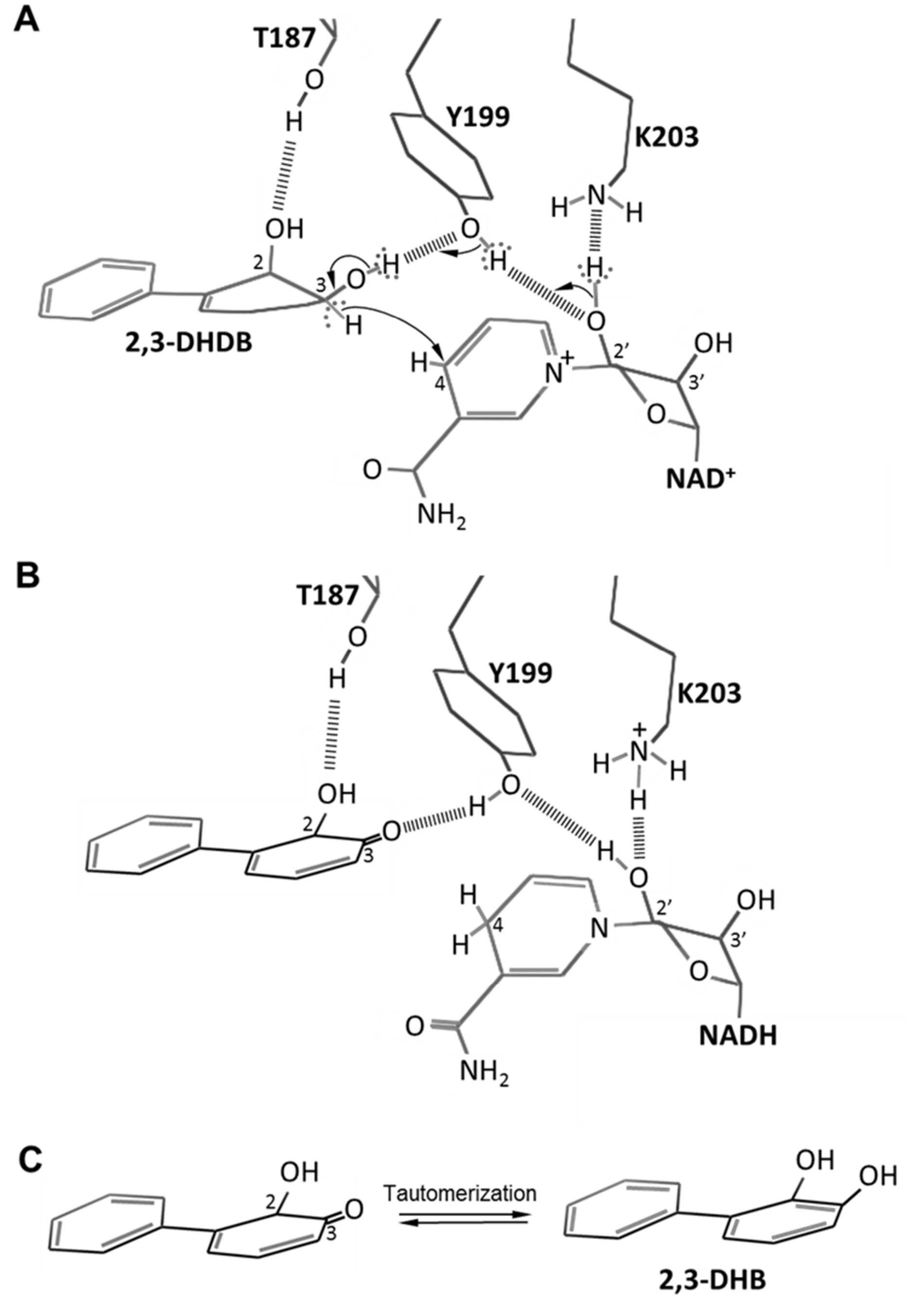

Figure S11. Catalytic mechanism proposed for poplar SDR57C. (*A*) A proton relay system involving Lys203 and NAD^+ ribose promotes proton subtraction from 2,3-DHDB by the catalytic base (Tyr199) as well as hydride transfer to NAD^+ nicotinamide. (*B*) As a result, an intermediate hydroxyketone and NADH are formed. (*C*) Keto-enol tautomerization of the intermediate ketone renders the re-aromatized catechol product, 2,3-DHB. This scheme reflects the exact geometry predicted for the ternary complex (Fig. 5).

Table S1. Peptide mass fingerprints for PCB-responsive polypeptides in poplar leaves.

Position in *P. trichocarpa* XP_002311602[a]	Observed mass	Predicted mass	Mass difference	Missed cuts	Peptide sequence[b]
37 – 49	1200.68	1200.65	0.03	0	VALVTGGDSGIGR
50 – 73	2732.34	2732.29	0.05	0	SVCYHFALEGATVAFTYVQGIEDR
87 – 108	2394.09	2394.05	0.04	1	SSDAEDPIAIATDVSSEEDCKR
120 – 146	3166.67	3166.63	0.04	0	IDILVNNAGVQHYTNLVEEITEEWL VR
147 – 160	1799.94	1799.91	0.03	1	LFRTNIFGYFFMTK
150 -160	1383.68	1383.65	0.03	0	TNIFGYFFMTK

[a] Spots 1 and 2 in Fig. 1*A* were subjected to MALDI-TOF analysis after trypsin digestion. Results were essentially identical (spot 1 is shown). The closest match in MASCOT searches was a SDR enzyme from *P. trichocarpa*, NCBI locus XP_002311602.

[b] Missed cleavages (underlined) are not unlikely for trypsin when P1' is Arg, as in peptide 87-108, or P2 is Phe, as in peptide 147-160 (2). Trypsin cleavage site: P2-P1[Arg/Lys]-↓-P1′-P2′.

Table S2. Primers used in this study.

qRT-PCR

Primer name[a]	*Sequence*	*Gene ID*
P. tremula* x *P. alba		
SDR57C-F	GCTCTGGTGACTGGAGGG	Potri.008G14920
SDR57C-R	CCTTCAGTAGCATCTTTAGGG	
Populus reference genes[b]		
18S rRNA-F	GATTCTATGGGTGGTGGTGC	
18S rRNA-R	CAGGCTGAGGTCTCGTTCG	
UBQ7-F	GGAACGGGTTGAGGAGAAAGAAG	
UBQ7-R	GCAAGAACAAGATGAAGCACAGAGC	
Arabidopsis reference genes		
18S rRNA-F	TCAACTTTCGATGGTAGGATAGTG	At3g41768
18S rRNA-R	CCGTGTCAGGATTGGGTAATT	
ACT8-F	GGTCGTACAACCGGTATTGT	At1g49240
ACT8-R	GAAGAGCATACCCCTCGTA	
CYPs		
At71A20-F	TCGAGCTTGAGAAAAGTGCC	At4g13310
At71A20-R	ATCTTGTGGTGAACTTGAACG	
At82C2-F	CCGAGGGTTTATATGGAGCC	At4g31970
At82C2-R	AGACCTAAATGAAGCACTTGC	
At710A1-F	GATGAAGTATACACGCTCCG	At2g34500
At710A1-R	GAACGAGGAGTCGAAAACCG	
At707A2-F	ATATGATGGCTACTTGATCCC	At2g29090
At707A2-R	GAATGGCATGTACGTGTAAGG	
At706A6-F	GTTTCTGACTTTTTCCCACGG	At4g12320
At706A6-R	TTCACCATCATCACCATCCC	
At77B1-F	GTTTCTGACTTTTTCCCACGG	At1g11600
At77B1-R	TTCACCATCATCACCATCCC	
EHs		
AtEH1-F	TTCCTACACGAAGAAAACCCT	At3g05600
AtEH1-R	TGAGCTTCATAATGCCAAGTC	
AtSEH-F	TTCCTACACGAAGAAAACCCT	At2g26740
AtSEH-R	GTCAAATAATATGCACGCATCC	
AtabHSP1-F	TTCCTACACGAAGAAAACCCT	At4g15960
AtabHSP1-R	CGGTTTACTCCGATTTTCCC	
AtabHSP2-F	TTCCTACACGAAGAAAACCCT	At2g26750
AabHSP2-F	CACATGATATGCACGCACCC	
AtabHSP3-F	GTGGTTGGACATGATTGGGG	At4g15955
AtabHSP3-F	TTGGTTTCCGTTTAGGATTCC	
AtabHSP4-F	CATCCTTGTAGTAGTTCTCCG	At4g02340
AtabHSP4-F	GATATATTGAACTGGAGATGGC	
Other genes		
AtHPPD-F	GCAGACGACGTTGGAACCG	At1g06570
AtHPPD-R	ATGTCTTCACTCATCAGAGCC	

AtSDR57C-F	GCATTATCAACACCACTTCGG	At1g54870
AtSDR57C-R	CCATATTGGACCAGGAGCC	

PCR-based cloning for expression of *P. tremula* SDR57C in *E. coli*

Primer name	*Sequence*[c]
SacI-SDR-F	TAAGAGCTCGAGAATGGAAGAACAAAGAAAACC
KpnI-SDR-R	CATGGTACCGAATTAACCATTTATAATCGTACC
attB1- SDR57C-F	AAAAAGCAGGCTAGAAGGAAAACAAACAATGGAAGAAC
attB2-SDR57C-R	AAGAAAGCTGGGTGATCAATTAACCATTTATAATCG
nested-attB1-F	GGGGACAAGTTTGTACAAAAAAGCAGGCTAG
nested-attB2-R	GGGGACCACTTTGTACAAGAAAGCTGGGTG
ApaI-SDR-F	CAGAAGGGCCCATTAATACGACTCACTATAGGG
SDR-EcoRV-R	AGCTCGATATCAATTAACCATTTATAATCGTACCC

PCR-based cloning for expression of *P. tremula* SDR57C in *Acidovorax*

Primer name	*Sequence*[c]
KpnI-SDR-F	CGGGGTACCAACAATGGAAGAACAAAGAAAACC
SacI-SDR-R	TCCGAGCTCGAATTAACCATTTATAATCGTACC

[a] F, forward primer. R, reverse primer. SDR, short-chain dehydrogenase reductase. UBQ, ubiquitin. ACT, actin. CYP, cytochrome P450. EH, epoxide hydrolase. SEH, soluble EH. abHSP, α/β-hydrolase superfamily protein. HPPD, 4-hydroxyphenylpyruvate dioxygenase.
[b] Primer pairs for *Populus* reference genes were designed as described (3).
[c] Shaded sequences: green, start codons; yellow, stop codons; blue, restriction sites; pink, *T7* promoter; grey, ribosome binding site for bacterial expression; orange, untranslated sequences from the *SDR57C* gene from *P. trichocarpa*. The *attB* sites for Gateway cloning are underlined.

SI Appendix References

1. B. Persson, Y. Kallberg, Classification and nomenclature of the superfamily of short-chain dehydrogenases /reductases (SDRs). *Chem. Biol. Interact*. **202**, 111–115 (2013).
2. J. A. Siepen, E. J. Keevil, D. Knight, S. J. Hubbard, Prediction of missed cleavage sites in tryptic peptides aids protein identification in proteomics. *J. Proteome Res.* **6**, 399–408 (2007).
3. E. A. Pettengill, C. Parmentier-Line, G. D. Coleman, Evaluation of qPCR reference genes in two genotypes of *Populus* for use in photoperiod and low-temperature studies. *BMC Res Notes* **5**, 366 (2012).
4. J. Boruc et al., TPX2-LIKE PROTEIN3 is the primary activator of α-aurora kinases and is essential for embryogenesis. *Plant Physiol*. **180**,1389–1405 (2019).
5. M. Heinig, D. Frishman, STRIDE: a Web server for secondary structure assignment from known atomic coordinates of proteins. *Nucl. Acids Res.* **32**, W500-W502 (2004).
6. M. Hülsmeyer et al., Crystal structure of cis-biphenyl-2,3-dihydrodiol-2,3-dehydrogenase from a PCB degrader at 2.0 Å resolution. *Prot. Sci*. **7**, 1286–1293 (1998).
7. M. E. Kovach et al., Four new derivatives of the broad-host-range cloning vector pBBR1MCS, carrying different antibiotic-resistance cassettes. *Gene* **166**, 175–176 (1995).
8. Y. Ohtsubo, Y. Nagata, K. Kimbara, M. Takagi, A. Ohta, Expression of the bph genes involved in biphenyl/PCB degradation in *Pseudomonas* sp. KKS102 induced by the biphenyl degradation intermediate, 2-hydroxy-6-oxo-6-phenylhexa-2,4-dienoic acid. *Gene* **256**, 223–228 (2000).
